# NF-κB signaling activation and roles in thyroid cancers: implication of MAP3K14/NIK

**DOI:** 10.1038/s41389-023-00496-w

**Published:** 2023-11-16

**Authors:** Françoise Cormier, Selma Housni, Florent Dumont, Mélodie Villard, Béatrix Cochand-Priollet, Françoise Mercier-Nomé, Karine Perlemoine, Jérôme Bertherat, Lionel Groussin

**Affiliations:** 1 Université Paris Cité, INSERM U1016, CNRS UMR8104, Institut Cochin, F-75014 Paris, France; 2 Service de Pathologie, Assistance Publique-Hopitaux de Paris, Hopital Cochin, Université Paris Cité, F-75014 Paris, France; 3 UMS IPSIT, Université Paris-Saclay, INSERM, CNRS, F-91400 Orsay, France; 4 Service d’Endocrinologie, Cochin AP-HP Centre, F-75014 Paris, France; 5 Present Address: Service de Médecine Nucléaire, Assistance Publique-Hopitaux de Paris, Hopital Pitié-Salpêtrière, F-75013 Paris, France; 6 Present Address: UMS IPSIT, Université Paris-Saclay, INSERM, CNRS, F-91400 Orsay, France

**Keywords:** Cell biology, Cancer

## Abstract

Among follicular-derived thyroid cancers (TC), those with aggressive behavior and resistance to current treatments display poor prognosis. NF-κB signaling pathways are involved in tumor progression of various cancers. Here, we finely characterize the NF-κB pathways and their involvement in TC. By using immunoblot and gel shift assays, we demonstrated that both classical and alternative NF-κB pathways are activated in ten TC-derived cell lines, leading to activated RelA/p50 and RelB/p50 NF-κB dimers. By analyzing the RNAseq data of the large papillary thyroid carcinoma (PTC) cohort from The Cancer Genome Atlas (TCGA) project, we identified a tumor progression-related NF-κB signature in BRAF^V600E^ mutated-PTCs. That corroborated with the role of RelA and RelB in cell migration and invasion processes that we demonstrated specifically in BRAF^V600E^ mutated-cell lines, together with their role in the control of expression of genes implicated in invasiveness (MMP1, PLAU, LCN2 and LGALS3). We also identified NF-κB-inducing kinase (NIK) as a novel actor of the constitutive activation of the NF-κB pathways in TC-derived cell lines. Finally, its implication in invasiveness and its overexpression in PTC samples make NIK a potential therapeutic target for advanced TC treatment.

## Introduction

Follicular-derived thyroid cancers (TC) are increasing in incidence in all countries over the five last decades [1]. More than 90% are differentiated types, mostly papillary thyroid cancer (PTC) and display favorable prognosis with a 5-year survival of 98%. However, the 10-year survival rate declines to 15–20% in patients with aggressive disease [2]. The more aggressive poorly differentiated TC (PDTC) or anaplastic (undifferentiated) TC (ATC) account for 5% of TC but for 40% of TC-related deaths. The mean survival of ATC patients is less than one year [3]. Thus the recurrent and metastatic PTC, PDTC and ATC form the aggressive “advanced thyroid cancer” group with a 5-year survival rate less than 50%. Encouraging therapeutic advances were accomplished with targeted therapies, but limited by frequent resistance and side-effects [4–6]. So the development of more accurate and personalized targeted therapies is still necessary. A deeper knowledge of molecular mechanisms involved in TC progression is required to identify novel biomarkers and therapeutic targets.

Targeting metastasis remains a challenge to improve cancer treatments [7]. The tumor progression toward the metastatic stage requires the epithelial-mesenchymal transition that involves chemokines, matrix metalloproteinases (MMPs), proteases, extracellular matrix proteins and cytoskeletal proteins. By regulating their expression, NF-κB transcription factors are implicated in metastatic progression of numerous solid tumors. So targeting NF-κB signaling represents a promising therapeutic strategy to control tumor progression [8].

The NF-κB/Rel family comprises five members, RelA/p65, RelB, c-Rel, NF-κB1/p105 (precursor of p50) and NF-κB2/p100 (precursor of p52). They act in homo- or heterodimers to activate target genes through binding on κB sites in their promoter. In the inactivated state, the NF-κB dimers are sequestered in the cytoplasm in complexes with one protein of the IκB (inhibitor of κB) family, such as IκBα, p105 or p100. Their activation may proceed through two main pathways [9]. In the classical pathway, signaling events end up at the activation of the IκB kinase β (IKKβ) in a complex with IKKα and IKKγ/ΝΕΜΟ. Activated IKKβ phosphorylates the IκB proteins, p105 and IκBα, that leads to their ubiquitynation and proteosomal degradation, allowing the release and the nuclear translocation of NF-κB dimers, mainly RelA/p50 and cRel/p50. The alternative pathway is dependent on the stabilization and activation of the MAP3K14 also named NF-κB Inducing Kinase (NIK) which in turn phosphorylates and activates IKKα. That leads to the phosphorylation and degradation of the IκB protein p100, allowing the release and nuclear translocation of RelB/p50 or RelB/p52 dimers.

Some studies pointed out the involvement of the NF-κB factors in neoplastic properties of TC, such as cell division, apoptosis resistance, invasiveness, cancer stem-cell properties and resistance to cancer therapy [10, 11]. Nevertheless their conflicting results did not make possible to definitively conclude how NF-κB factors contribute to TC development. This could reflect heterogeneity of thyroid cancers in NF-κB activation in relation with clinical/mutational features which remains to identify.

The aim of this study was to fully characterize NF-κB signaling in TC, identify upstream activator(s) and precise its physiopathological role. This work provides the first demonstration of alternative NF-κB pathway activation in TC. We also demonstrate the role of NF-κB pathways in invasive properties specifically in BRAF-mutated cell lines. The metastasis-related NF-κB signature that we identified in the BRAF-mutated PTC cohort from the TCGA database corroborates the role of NF-κB in invasiveness of BRAF^V660E^ TC-derived cell lines and supplies NF-κB activation biomarkers. Furthermore, we identified MAP3K14/NIK as an upstream activator of the NF-κB pathways in TC, that makes it a novel actor of thyroid carcinogenesis.

## Results

### The classical and alternative NF-κB pathways are constitutively activated in thyroid cell lines

**Fig. 1:**
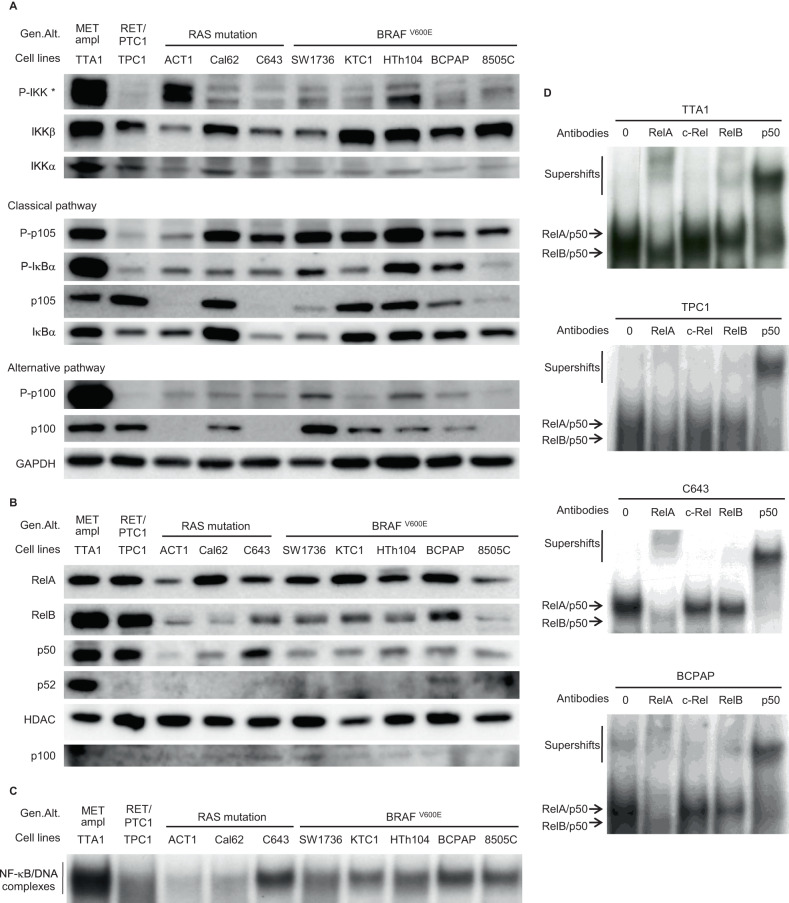
The NF-κB pathways are constitutively activated in thyroid carcinoma cell lines. **A** The expression and site-specific phosphorylation of components of the classical and alternative NF-κB pathways were analyzed in immunoblot assay of cytoplasmic proteins with appropriate antibodies. GAPDH level was used as a protein loading control. *The P-IKK upper band corresponds to IKKβ and the lower band to IKKα. **B** The nuclear localization of RelA, RelB, p50 and p52 proteins was analyzed in immunoblot assay of nuclear protein extracts with the appropriate antibodies. HDAC level was used as a nuclear protein loading control, and p100 level was led as an indicator of nuclear extraction quality. **C** The baseline NF-κB activity in total protein extracts of thyroid carcinoma cell lines was assessed in gel shift experiment. **D** The NF-κB subunits contributing to the NF-κB activity in 4 thyroid carcinoma cell lines were identified in supershift experiments with anti-RelA, -c-Rel, -RelB and -p50 antibodies. Data of representative experiments are shown. Gen.Alt. : Genomic Alteration. ampl: amplification.

We analyzed the NF-κB pathways activation in ten TC-derived cell lines, of which nine harbor one of the most frequent genomic alterations involved in TC (five with the BRAF T1999A mutation, three with a RAS mutation and the TTA1 cell line with a RET-PTC rearrangement). We first examined the baseline activation of the classical and alternative NF-κB pathways by analyzing their specific signaling events (Fig. 1A). The central regulating IKKβ and IKKα kinases were diversely activated, as demonstrated by their various phosphorylation levels. The classical pathway was significantly activated in most of the cell lines, as indicated by the high phosphorylation level of the IκB proteins IκBα and p105/NF-κB1. The high p100/NF-κB2 phosphorylation in the TTA1 cell line (harboring a c-MET amplification) indicated a stronger activation of the alternative pathway in this cell line, in comparison with the other ones. The RelA and RelB nuclear localization also evidenced the activation of both classical and alternative pathways, together with the nuclear localization of their p50 partner (Fig. 1B). Nuclear p52 was barely detected except in the TTA1 cell line showing a more significant expression (Fig. 1B). No c-rel nuclear localization has been evidenced in any of these cell lines (data not shown). The analysis of DNA-binding capacities in gel shift assays clearly demonstrated the activation of NF-κB factors in all these cell lines, albeit of variable intensity (Fig. 1C). The strongest NF-κB DNA-binding capacity which characterized the TTA1 cell line is in agreement with the strong activation of the NF-κB pathways, as demonstrated in Fig. 1A. We further explored classical and alternative pathways activation by identifying the subunits of NF-κB dimers complexed with the DNA probe. We carried out supershift experiments in cell lines which displayed the highest NF-κB activation, namely TTA1, TPC1, C643 and BCPAP (Fig. 1D). The NF-κB-DNA complexes were supershifted and/or reduced by anti-RelA, anti-RelB and anti-p50 antibodies but not by anti-c-rel antibody, thus demonstrating the activation of RelA/p50 and RelB/p50 dimers. Altogether, these results demonstrated the constitutive activation of both classical and alternative NF-κB pathways in ten different thyroid carcinoma cell lines. We noticed a high NF-κB activity in the five BRAF-mutated cell lines, in the MET-amplified cell line and in the RET-PTC-cell line, and in only one out of the three RAS mutated-cell lines.

### RelA and RelB are required for migration and invasion potential of BRAF-mutated cell lines

We next investigated the cellular role of NF-κB factors in TC cell lines in relation with their mutational status. We selected the BCPAP and 8505C cell lines as BRAF^V600E^ expressing-cell line, for comparison with cell lines displaying another genomic alteration with similar NF-κB activity, the TTA1 and C643 cell lines.

**Fig. 2:**
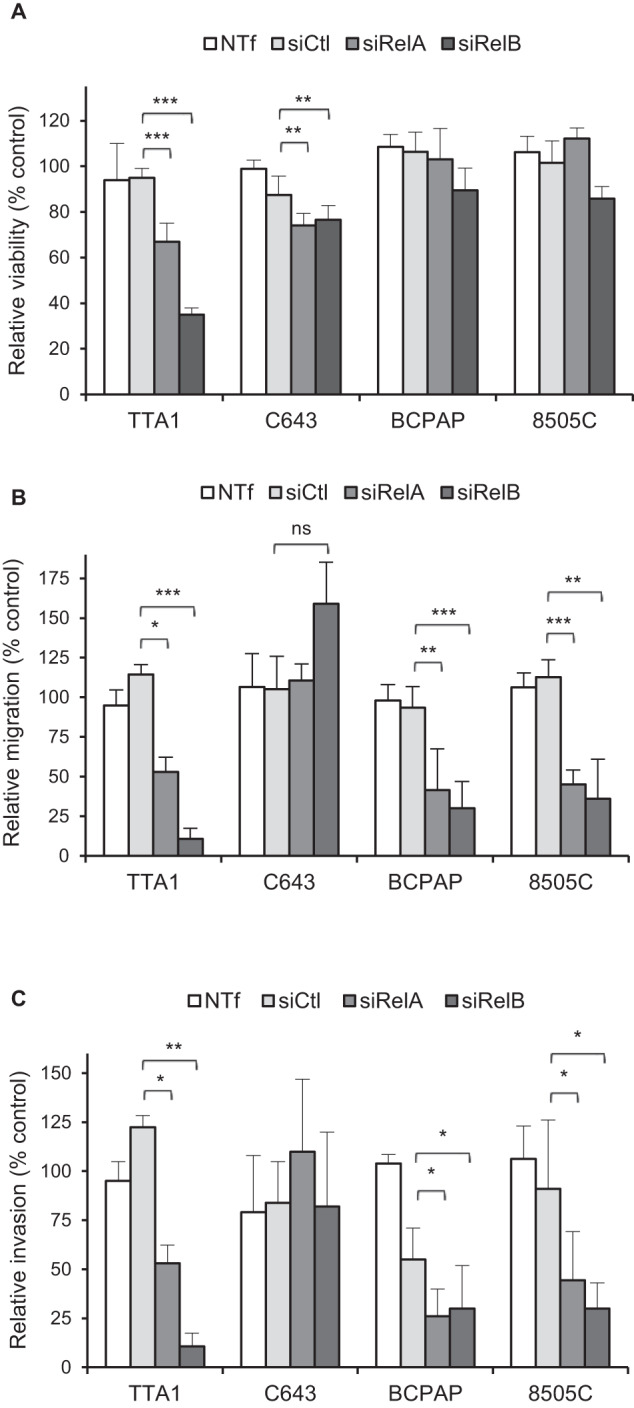
RelA and RelB are differently implicated in the growth, motile and invasive potentials of thyroid cell lines. After transfection with control siRNA (siCtl), RelA siRNA (siRelA), or RelB siRNA (siRelB), the indicated cell lines were plated for viability (**A**), migration (**B**) or invasion (**C**) assay. (**A**) The mean results of relative viability ±SD compared to non transfected (NTf) cells of eight replicates in two independent experiments are reported. **B**, **C** The results are means ± SD of relative motility or invasion compared to NTf cells in two or three independent migration and invasion experiments carried out in duplicate. ns: not significative.

As shown in Fig. 2A and S1A, the siRNA-mediated RelA-, or RelB-decreased expression did not significantly reduce the growth of the BCPAP and 8505C cell lines, but slightly the one of the C643 cell line. Conversely in the TTA1 cell line, the growth capacity decrease by half and quart after RelA- or RelB-downregulation, respectively, indicated the significant proliferative role of NF-κB factors in this cell line.

We next explored the NF-κB involvement in the motility and invasiveness (Fig. 2B, C and S1B). Reduced RelA or RelB expression strongly reduced the migration and invasion capacities of the TTA1 cell line and the two BRAF-mutated cell lines (BCPAP and 8505C), while not significantly affecting the RAS-mutated C643 cell line. The migration and invasion potential was reduced from 40 to 20 % compared to control cells. Notably the RelB-reduced expression led to a tenfold reduction of the migration capacity of TTA1 cells.

### A tumor progression-related NF-κB signature distinguishes BRAF-mutated PTC

**Fig. 3:**
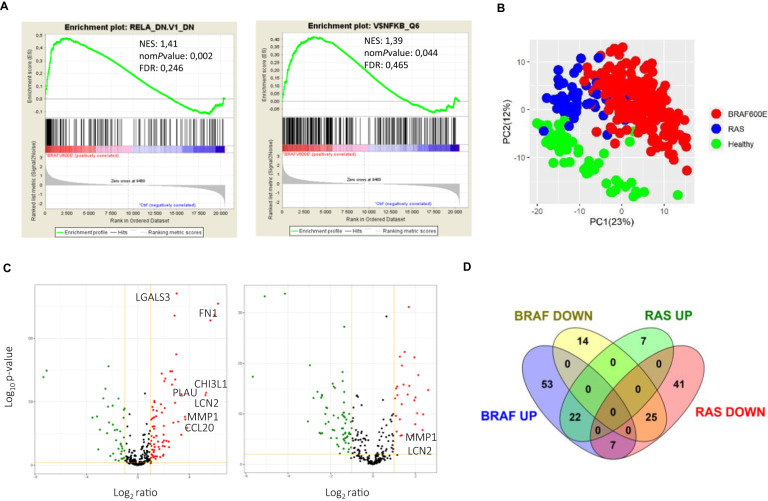
The PTC transcriptional program includes NF-κB signatures. **A** GSEA enrichment plots for the oncogenic signature RelA_DN.V1_DN and for the gene set NFKB_Q6 (set of genes with the transcription factor binding site V$NFKB_6 which is bound by NF-κB factors members in the regions up to 4 kb around their transcription starting sites) in BRAF-mutated PTCs. **B** Principal component analysis of the NF-κB target genes set expression in BRAF-, RAS- mutated PTCs and healthy thyroid tissues. **C** Volcano plots of the differential expression of the NF-κB target genes in BRAF- (left panel) and RAS- (right panel) mutated PTCs. Red and green dots represent significantly upregulated or downregulated genes, respectively, and gray dots represent not significantly deregulated genes (*P* value < 0.01 and fc >2). Selected genes from the tumor-progression associated signature are highlighted. **D** Venn diagram representation of the differentially expressed NF-κB target genes in BRAF-mutated PTCs and RAS-mutated PTCs.

We further explored NF-κB activity together with its oncogenic significance in TC by searching for thyroid tumorigenesis-associated NF-κB signatures through the comparison of the transcriptional programs of PTCs and healthy thyroid tissues. For that, we used the RNAseq data of the large PTC cohort from TCGA project [12]. First, a Gene Set Enrichment Analysis (GSEA) revealed that a NF-κB-related oncogenic signature (RelA_DN.V1_DN) and a set of genes with NF-κB binding sites around their transcription starting sites were significantly enriched in BRAF-mutated PTCs (Fig. 3A), and not in RAS-mutated tumors. The analysis of the differential gene expression between PTCs and healthy tissues with “Ingenuity Pathway Analysis” (IPA) software identified the NF-κB signaling as activated in BRAF-mutated PTCs (Table 1), and not in RAS-mutated PTCs. Components of the NF-κB pathways (NF-κB proteins, IKKα, IKKβ and MAP3K14/NIK) were identified among the significantly activated upstream regulators only in BRAF-mutated PTCs. Sixteen upstream regulators signaling towards the NF-κB pathways, such as TNF and TNFR superfamilies members, interleukins or Toll-Like Receptors (TLR) were identified highly significantly activated in BRAF-mutated PTCs, compared to only one in RAS-mutated PTCs, TNFRSF21, a TNF receptor superfamily member (activation z-score:2; *p* value: 9,61.10^–2^).

**Table 1:** Ingenuity pathway analysis of NF-κB-related upstream regulators in BRAF-mutated PTC.

Upstream regulator	Molecule type	Activation z-score	*p* value
*TNF*	*cytokine*	*7.321*	*4.33E–50*
*LPS*	*chemical drug*	*6.294*	*5.43E–30*
*IL6*	*cytokine*	*3.813*	*6.16E–27*
*IL1B*	*cytokine*	*4.503*	*2.62E–26*
*IL1*	*group*	*4.485*	*6.81E–18*
**IKBΚB / IKKβ**	**kinase**	**2.079**	**5.54E–17**
*IL13*	*cytokine*	*2.308*	*9.62E–17*
**NFκB (complex)**	**complex**	**4.343**	**.398E–15**
**CHUK / IKKα**	**kinase**	**2.591**	**3.54E–14**
*PI3K (complex)*	*complex*	*2.733*	*1.33E–12*
**NFΚB1**	**transcription regulator**	**2.294**	**3.99E–12**
*IL1A*	*cytokine*	*4.207*	*4.53E–11*
*E. coli B5 LPS*	*chemical - endogenous non-mammalian*	*3.601*	*7.91E–08*
**REL**	**transcription regulator**	**2.279**	**3.92E–06**
**NFκB1-RelA**	**complex**	**3.225**	**1.72E–05**
*TLR3*	*transmembrane receptor*	*2.357*	*4.62E–05*
*TNFSF14*	*cytokine*	*3.023*	*7.08E–05*
*TLR4*	*transmembrane receptor*	*2.379*	*7.79E–05*
*TNFRSF13B*	*transmembrane receptor*	*2.043*	*2.36E–03*
*TNFRSF1B*	*transmembrane receptor*	*2.575*	*4.41E–03*
**NFκB (family)**	**group**	**2.151**	**7.72E–03**
*TNFRSF14*	*transmembrane receptor*	*2*	*1.08E–02*
**NFκB-RelA**	**complex**	**2.185**	**2.14E–02**
*Lymphotoxin*	*complex*	*2.219*	*7.30E–02*
**MAP3K14/NIK**	**kinase**	**3.046**	**1.22E–01**

The significantly activated NF-κB-related upstream regulators predicted based on genes that were significantly deregulated in BRAF-mutated PTCs compared to healthy thyroid tissue are shown. The 9 upstream regulators which are components of the NF-κB pathways are indicated in bold. The 16 upstream regulators signaling toward the NF-κB pathways are indicated in italic. The z-score >2 indicates the possible activation of the molecule based on prior knowledge stored in the Ingenuity Knowledge Base. The *p* value indicates the significance based on the overlap between dataset genes and known targets regulated by the molecule.

**Fig. 4:**
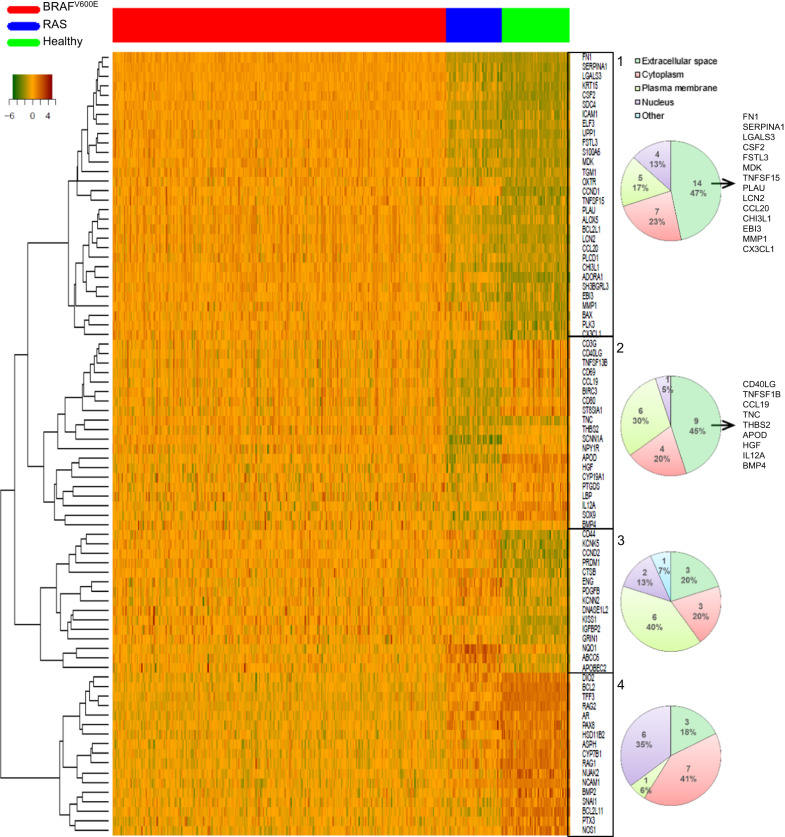
A tumor progression NF-κB related signature characterizes the BRAF-mutated PTCs. Heatmap for relative expression of the top 50 more significantly differentially expressed NF-κB target genes between BRAF-mutated PTCs, or RAS-mutated PTCs and healthy thyroid tissues in BRAF-mutated PTCs (*N* = 286), RAS-mutated PTCs (*N* = 48) and healthy thyroid tissues (*N* = 58) from the TCGA database. Pie charts represent the distribution of the localization of gene products comprised in clusters 1, 2, 3 and 4, as indicated. The genes from clusters 1 and 2 whose the product is expressed in the extracellular space are listed in right.

To identify NF-κB signatures in PTC, we analyzed the differential expression of a set of 363 validated NF-κB target genes (Table S1) between PTCs and healthy thyroid tissues. A principal component analysis revealed a clear segregation between BRAF-, RAS-mutated PTCs and healthy tissues (Fig. 3B). Ingenuity pathway analysis showed that 121 genes (33%) from the NF-κB target genes set were highly and significantly deregulated in BRAF-PTCs (*p* value = 1.54 × 10^–21^) and 102 (28%) in RAS-tumors (p-value = 1.34 × 10^–21^) (Tables S2, S3). The differentially expressed NF-κB target genes were mainly upregulated (68%), more intensively (from 75 to 2.6 in the top 50) and more significantly (*p* values from 5.11 × 10^–161^) in the BRAF-PTCs compared to the RAS-PTCs in which most were downregulated (73%) (Figs. 3C, D and S2, Tables S2, S3). Unsupervised hierarchical clustering of the 50 more significantly differentially expressed NF-κB target genes in the BRAF- or RAS-PTCs (Tables 2, 3) identified 4 genes subsets which segregated the BRAF-PTCs, the RAS-PTCs and the healthy thyroid tissues (Fig. 4). Interestingly, two clusters (cluster 1 and 2) mainly comprised genes whose products are localized in the extracellular space (Fig. 4). And most of the genes from cluster 1 are well known for their implication in invasiveness such as Fibronectin 1 (FN1), Alpha-1-Antitrypsin (SERPINA1), Galectin 3 (LGALS3), Plasminogen Activator Urokinase (PLAU), Lipocalin (LCN2), C-C motif Chemokine Ligand 20 (CCL20), Chitinase 3 Like 1 (CHI3L1), and Matrix Metallopeptidase 1 (MMP1). Thus, these analyses showed that BRAF-PTCs and RAS-PTCs distinguish from healthy thyroid tissues and from each other by their NF-κB-related transcriptional program, with a stronger and specific NF-κB signature in BRAF-PTCs related to cell invasion process by upregulating components of the extracellular space.

**Table 2:** List of the 50 more significantly deregulated genes from the set of 363 validated NF-κB target genes in BRAF-mutated PTCs compared to healthy thyroid tissues.

Symbol	Gene name	Exp *p* value	Exp fold change	Location
SERPINA1	serpin family A member 1	5.11E–161	38.981	Extracellular Space
LGALS3	galectin 3	4.37E–136	8.100	Extracellular Space
FN1	fibronectin 1	5.28E–128	75.316	Extracellular Space
CSF2	colony stimulating factor 2	4.98E–119	62.080	Extracellular Space
SDC4	syndecan 4	1.27E–118	7.192	Plasma Membrane
KRT15	keratin 15	7.36E–115	49.841	Cytoplasm
ADORA1	adenosine A1 receptor	3.63E–88	7.889	Plasma Membrane
BCL2	BCL2 apoptosis regulator	1.08E–78	–4.886	Cytoplasm
RAG2	recombination activating 2	3.24E–75	–136.707	Nucleus
ICAM1	intercellular adhesion molecule 1	1.04E–74	6.343	Plasma Membrane
UPP1	uridine phosphorylase 1	1.05E–74	5.739	Cytoplasm
CCND1	cyclin D1	5.67E–73	3.587	Nucleus
TFF3	trefoil factor 3	3.95E–70	–162.441	Extracellular Space
FSTL3	follistatin like 3	5.67E–69	4.372	Extracellular Space
MDK	midkine	3.02E–68	7.461	Extracellular Space
S100A6	S100 calcium binding protein A6	5.26E–67	4.136	Cytoplasm
ELF3	E74 like ETS transcription factor 3	3.37E–64	6.295	Nucleus
RAG1	recombination activating 1	5.86E–61	–5.747	Nucleus
CHI3L1	chitinase 3 like 1	8.31E–58	39.441	Extracellular Space
PLAU	plasminogen activator. urokinase	1.22E–56	10.471	Extracellular Space
LCN2	lipocalin 2	5.59E–56	38.034	Extracellular Space
DIO2	iodothyronine deiodinase 2	2.12E–53	–6.443	Cytoplasm
NUAK2	NUAK family kinase 2	3.62E–53	–2.847	Nucleus
BCL2L1	BCL2 like 1	2.69E–51	2.428	Cytoplasm
CCND2	cyclin D2	3.17E–50	2.452	Nucleus
EBI3	Epstein-Barr virus induced 3	8.61E–50	7.417	Extracellular Space
TGM1	transglutaminase 1	2.93E–49	4.756	Plasma Membrane
OXTR	oxytocin receptor	1.10E–48	3.445	Plasma Membrane
CYP7B1	cytochrome P450 family 7 subfamily B member 1	1.27E–47	–3.891	Cytoplasm
AR	androgen receptor	7.26E–47	–5.004	Nucleus
KCNK5	potassium two pore domain channel subfamily K member 5	1.14E–46	2.186	Plasma Membrane
TNFSF15	TNF superfamily member 15	9.18E–46	4.235	Extracellular Space
BCL2L11	BCL2 like 11	1.89E–45	–2.033	Cytoplasm
PAX8	paired box 8	2.18E–44	–2.130	Nucleus
BAX	BCL2 associated X. apoptosis regulator	2.84E–44	2.289	Cytoplasm
PLK3	polo like kinase 3	1.20E–43	2.752	Nucleus
PRDM1	PRSET domain 1	3.09E–42	3.631	Nucleus
CX3CL1	C-X3-C motif chemokine ligand 1	2.60E–39	2.665	Extracellular Space
NOS1	nitric oxide synthase 1	3.73E–39	–9.027	Cytoplasm
MMP1	matrix metallopeptidase 1	6.55E–39	12.575	Extracellular Space
ALOX5	arachidonate 5-lipoxygenase	7.87E–39	12.364	Cytoplasm
SH3BGRL3	SH3 domain binding glutamate rich protein like 3	1.61E–38	2.407	Nucleus
NCAM1	neural cell adhesion molecule 1	2.98E–38	–11.542	Plasma Membrane
ASPH	aspartate beta-hydroxylase	3.61E–38	–2.158	Cytoplasm
CD44	CD44 molecule (Indian blood group)	8.69E–38	2.205	Plasma Membrane
CCL20	C-C motif chemokine ligand 20	7.49E–37	12.851	Extracellular Space
PLCD1	phospholipase C delta 1	9.94E–37	2.047	Cytoplasm
KISS1	KiSS-1 metastasis suppressor	4.70E–35	8.735	Cytoplasm
HSD11B2	hydroxysteroid 11-beta dehydrogenase 2	1.76E–34	–4.732	Cytoplasm
TNC	tenascin	2.95E–34	6.754	Extracellular Space

**Table 3:** List of the 50 more significantly deregulated genes from the set of 363 validated NF-κB target genes in RAS-mutated PTCs compared to healthy thyroid tissues.

Symbol	Entrez gene name	Exp *p* value	Exp fold change	Location
TFF3	trefoil factor 3	1.70E–34	–17.687	Extracellular Space
APOD	apolipoprotein D	5.63E–34	–34.180	Extracellular Space
CCND1	cyclin D1	7.48E–32	3.224	Nucleus
ABCC6	ATP binding cassette subfamily C member 6	2.25E–29	2.622	Plasma Membrane
BCL2L11	BCL2 like 11	5.75E–28	–2.547	Cytoplasm
CD44	CD44 molecule (Indian blood group)	5.66E–23	2.821	Plasma Membrane
PDGFB	platelet derived growth factor subunit B	4.77E–22	2.409	Extracellular Space
ADORA1	adenosine A1 receptor	6.37E–22	3.870	Plasma Membrane
SCNN1A	sodium channel epithelial 1 subunit alpha	2.42E–20	–7.800	Plasma Membrane
CCND2	cyclin D2	2.86E–20	2.580	Nucleus
SOX9	SRY-box transcription factor 9	5.63E–19	–2.699	Nucleus
NQO1	NAD(P)H quinone dehydrogenase 1	2.22E–18	4.387	Cytoplasm
CCL19	C-C motif chemokine ligand 19	4.76E–18	–50.332	Extracellular Space
PTX3	pentraxin 3	3.26E–17	–2.686	Extracellular Space
SNAI1	snail family transcriptional repressor 1	1.08E–16	–3.728	Nucleus
ENG	endoglin	1.71E–16	2.716	Plasma Membrane
DNASE1L2	deoxyribonuclease 1 like 2	2.99E–16	3.267	Extracellular Space
KCNK5	potassium two pore domain channel subfamily K member 5	3.93E–16	2.213	Plasma Membrane
CD80	CD80 molecule	4.21E–16	–3.617	Plasma Membrane
HGF	hepatocyte growth factor	7.65E–16	–3.443	Extracellular Space
BAX	BCL2 associated X, apoptosis regulator	9.60E–16	2.129	Cytoplasm
CSF2	colony stimulating factor 2	2.02E–15	6.075	Extracellular Space
CD40LG	CD40 ligand	4.36E–15	–6.115	Extracellular Space
NUAK2	NUAK family kinase 2	4.74E–15	–2.554	Nucleus
PRDM1	PRSET domain 1	1.52E–14	3.106	Nucleus
NOS1	nitric oxide synthase 1	1.78E–14	–5.291	Cytoplasm
IGFBP2	insulin like growth factor binding protein 2	1.96E–14	2.219	Extracellular Space
ST8SIA1	ST8 alpha-N-acetyl-neuraminide alpha-2,8-sialyltransferase 1	2.79E–14	–5.281	Cytoplasm
APOBEC2	apolipoprotein B mRNA editing enzyme catalytic subunit 2	5.62E–14	4.000	Other
LBP	lipopolysaccharide binding protein	6.54E–14	–5.431	Plasma Membrane
PTGDS	prostaglandin D2 synthase	7.45E–14	–6.952	Cytoplasm
CTSB	cathepsin B	9.10E–14	2.061	Cytoplasm
KISS1	KiSS-1 metastasis suppressor	2.25E–13	3.864	Cytoplasm
IL12A	interleukin 12A	5.75E–13	–2.574	Extracellular Space
KCNN2	potassium calcium-activated channel subfamily N member 2	1.29E–12	2.559	Plasma Membrane
CD3G	CD3g molecule	5.70E-12	–4.609	Plasma Membrane
BIRC3	baculoviral IAP repeat containing 3	7.07E–12	–4.475	Cytoplasm
HSD11B2	hydroxysteroid 11-beta dehydrogenase 2	5.93E–11	–2.614	Cytoplasm
NPY1R	neuropeptide Y receptor Y1	5.93E–11	–2.572	Plasma Membrane
GRIN1	glutamate ionotropic receptor NMDA type subunit 1	7.47E–11	4.237	Plasma Membrane
BMP4	bone morphogenetic protein 4	1.04E–10	–2.173	Extracellular Space
CD69	CD69 molecule	1.09E–10	–4.744	Plasma Membrane
KRT15	keratin 15	1.28E–10	5.174	Cytoplasm
TNFSF13B	TNF superfamily member 13b	1.55E–10	–2.714	Extracellular Space
CYP19A1	cytochrome P450 family 19 subfamily A member 1	2.07E–10	–3.616	Cytoplasm
UPP1	uridine phosphorylase 1	2.50E–10	2.121	Cytoplasm
NCAM1	neural cell adhesion molecule 1	2.97E–10	–2.597	Plasma Membrane
THBS2	thrombospondin 2	4.98E–10	–2.499	Extracellular Space
BMP2	bone morphogenetic protein 2	6.20E–10	–3.120	Extracellular Space
TNFSF15	TNF superfamily member 15	1.20E–09	2.406	Extracellular Space

### RelA and RelB regulate genes from the tumor progression-related NF-κB signature

**Fig. 5:**
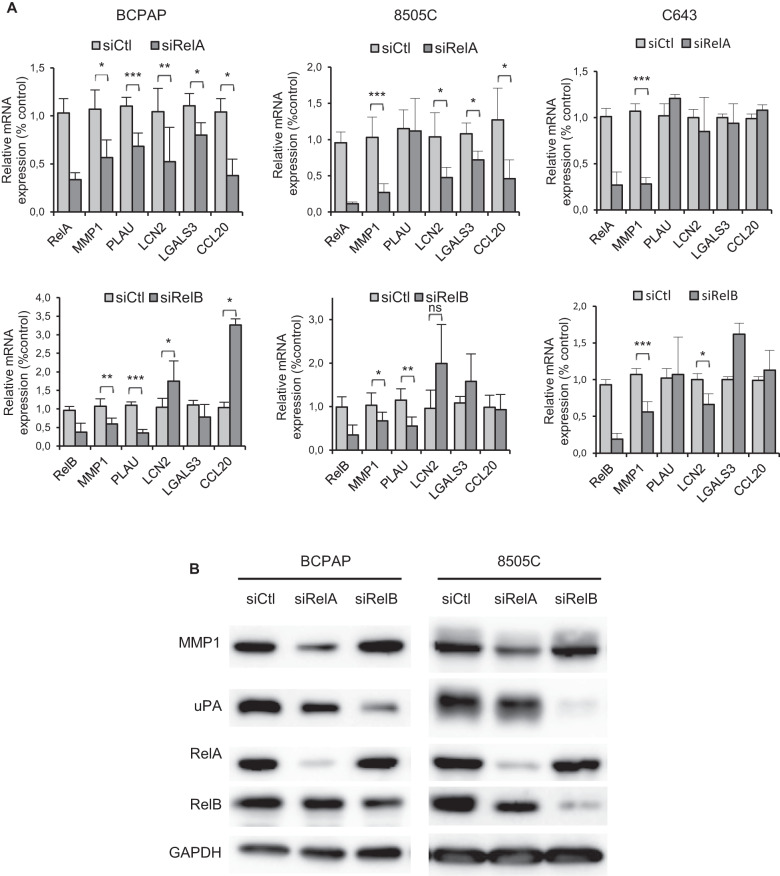
RelA and RelB regulate the expression of cell invasion-related genes of the NF-κB signature of BRAF-mutated PTCs. The BCPAP, 8505C and C643 cell lines were transfected with control siRNA (siCtl), RelA siRNA (siRelA), or RelB siRNA (siRelB). **A** Two days after transfection, mRNA expression of the indicated genes was analyzed by qRT-PCR. Results are means ± SD of relative mRNA expression level compared to control in four or five independent experiments. **B** Protein expression was analyzed in immunoblot assay with the appropriate antibodies. Data of representative experiments are shown.

We next checked whether genes of the tumor progression-related NF-κB signature evidenced in BRAF-PTCs were actually regulated by RelA or RelB in BRAF^V600E^ cell line models. The RelA downregulation induced a significant reduction of MMP1, LCN2, LGALS3 and CCL20 mRNA expression in both BCPAP and 8505C cell lines, and the one of PLAU only in the BCPAP cell line (Fig. 5A). The MMP1 and PLAU mRNA expression also required RelB expression, but, surprisingly, the LCN2, LGALS3 and CCL20 genes transcription were activated in one or two of these cell lines after RelB downregulation (Fig. 5A). Interestingly, the RelA or RelB downregulation didn’t significantly affect the expression of these genes in the C643 RAS-mutated cell line, except the one of MMP1 and LCN2. That corroborates with the finding that MMP1 and LCN2 was highly and significantly activated in both BRAF- and RAS-mutated PTCs, differently to PLAU, LGALS3 and CCL20 which were specifically activated in BRAF-PTCs (Fig. 3C, D, Tables 2, 3). The RelA downregulation severely decreased the MMP1 protein expression, while the expression of the product of PLAU, uPA (urokinase plasminogen activator), was strongly decreased by the RelB downregulation (Fig. 5B).

### NIK is implicated in the NF-κB activation and the cell invasion process in BRAF-mutated cell lines

**Fig. 6:**
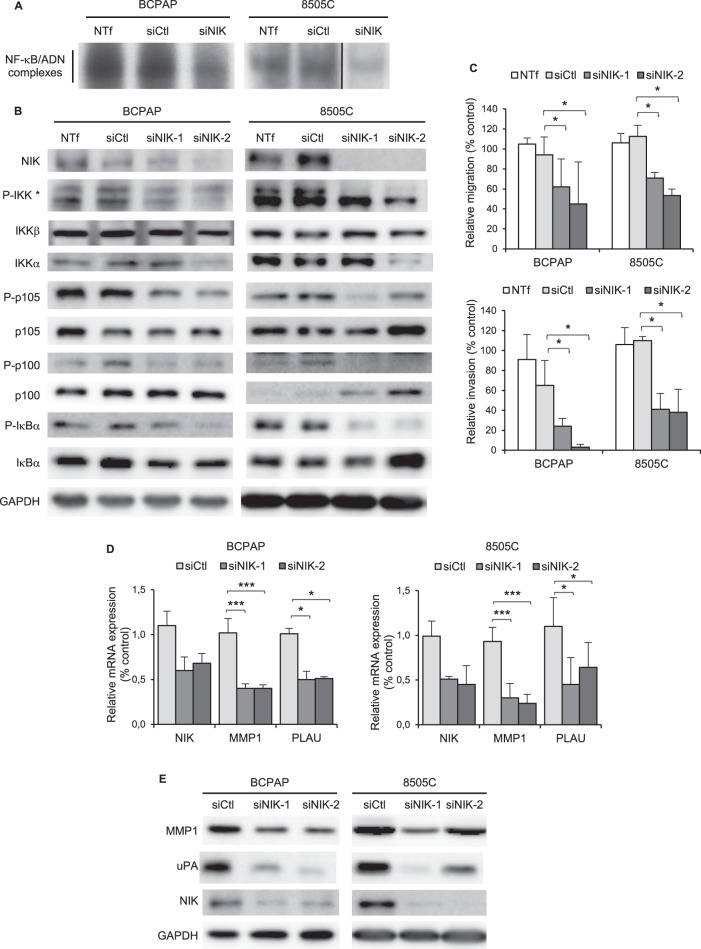
The MAP3K NIK contributes to the NF-κB pathways activation and regulates the cell invasion process in BRAF-mutated cell lines. The BCPAP and 8505C cell lines were transfected with control (siCtl) or NIK siRNA (siNIK, siNIK-1 or siNIK-2). Not transfected cells (NTf) were included as another negative control. **A** Two days after transfection, NF-κB activity analysis was performed in EMSA experiment as in Fig. 1C. **B**. Expression and site-specific phosphorylation of components of the classical and alternative NF-κB pathways were analyzed in immunoblotting assay of whole cell proteins. (*The P-IKK upper band corresponds to IKKβ and the lower band to IKKα). **C** Assessment of migration (upper panel) and invasion (bottom panel) capacities. The results are means ± SD of two independent migration and invasion experiments carried out in duplicated. **D** MMP1 and PLAU mRNA expression analysis were performed by qRT-PCR. The results are means ± SD of relative mRNA expression level compared to control in two or three experiments. **E** MMP1 and uPA protein expression analysis was analyzed in immunoblot assay. Data of representative experiments are shown in (**B**, **E**).

We next searched for upstream activators of the NF-κB pathways in BRAF ^V600E^ cell lines. The NF-κB activity in the BCPAP and 8505C cell lines was not dependent on the MAPK pathway activation, as shown by no-effect of the MEK1/2 pharmacological inhibitors UO126 and AZD6244 (Fig. S3). We demonstrated that the siRNA-mediated downregulation of MAP3K14/NIK clearly reduced the NF-κB activity, as detected in gel shift experiments (Fig. 6A, S4A). We further demonstrated the implication of NIK with the consequences of its invalidation on the NF-κB pathways. The downregulation of NIK in the BCPAP and 8505C cell lines led to more or less reduced IKKβ and IKKα phosphorylation (Fig. 6B, S4B). That was associated with a reduced p105, p100 and IκB phosphorylation (Fig. 6B), thus demonstrating that NIK is implicated in the activation of both classical and alternative NF-κB pathways in these cell lines.

We investigated whether targeting NIK could affect the migration and invasion potentials of BRAF-mutated cell lines. The NIK downregulation more intensively reduced the invasive properties of both BCPAP and 8505C cell lines than their migration potential (Fig. 6C, S4C). This functional impact of the NIK downregulation was associated with a reduced expression of MMP1 and PLAU mRNA (Fig. 6D) and their products (Fig. 6E).

### NIK is expressed and RelB is activated in BRAF-mutated PTCs

**Fig. 7:**
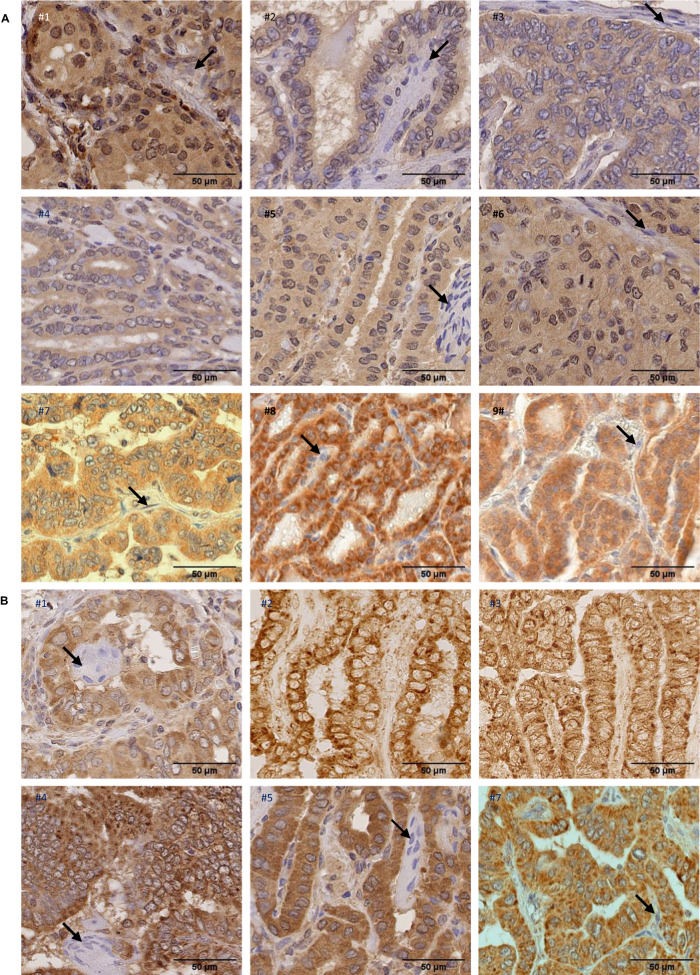
NIK and RelB expression in primary papillary thyroid cancer. **A** Paraffin-embedded sections of nine PTC samples were immunostained using anti-NIK antibody and counterstained with hematoxylin. Representative views showing positive immunoreactivity in neoplastic papillae compared to stromal cells (indicated by arrows) with no immunoreactivity, are presented. **B** Paraffin-embedded sections of six PTC samples were immunostained using anti-RelB antibody and counterstained with hematoxylin (samples #1, #4, #5 and #7) or only immunostained using anti-RelB antibody (samples #2 and #3). Representative views showing positive cytoplasmic and nuclear immunoreactivity in tumoral cells are presented. Nuclear immunoreactivity is indicated by a slight gray to brown staining of nuclei in samples #1, #4, #5 and #7, compared to nuclei multinucleated giant cells (indicated by arrows in #1, #4 and #5) or stromal cells (indicated by an arrow in #7) which displayed blue staining. No hematoxylin staining in samples #2 and 3 allowed to clearly detect RelB nuclear immunopositivity in tumoral cells through a slight peroxydase-positive staining.

The major mechanism by which NIK function is regulated is its stabilization through the inhibition of the ubiquitynation-mediated degradation [13]. Therefore a significant expression of NIK protein in tissue is indicative of its functionality. Tumor tissue samples from nine PTCs (Table S4) were subjected to NIK expression analysis by immunochemical staining. As illustrated in Fig. 7A, a significant NIK immunoreactivity was observed in the neoplastic papillae in all samples, while stromal cells in their central axis didn’t display staining. NIK was detected in cytoplasm, but we also noticed a slight nuclear staining, as reported by Birbach and coll [14].

The NF-κB activation in PTC tissue samples was previously reported through the nuclear localization of RelA, also observed in our samples (Fig. S5). Anti-RelB immunohistochemical staining of these samples allowed to also detect RelB in nuclei (Fig. 7B), thus confirming RelB activation that we demonstrated in cell lines with EMSA supershift experiments (Fig. 1D).

## Discussion

The standard therapeutic strategy for follicular-derived TC is efficient for the large majority of differentiated cancers. But treating patients with recurrence, progressive or aggressive disease remains a therapeutic challenge which calls for the in-depth knowledge of the molecular mechanisms involved in thyroid carcinogenesis.

Beside major oncogenic signaling such as the MAPK, PI3K-AKT or RTKs (Receptor Tyrosine Kinase) pathways, the classical NF-κB pathway was implicated in the pathophysiology of TC. The constitutive NF-κB DNA-binding activity associated with the increased expression of RelA was first demonstrated in PTC- and ATC-derived cell lines [15], followed by the nuclear localization of RelA in PTC, FTC and ATC tumor tissues [16–18]. Data about other NF-κB subunits were limited to the expression or the DNA-binding capacity of p50 in cell lines [15, 19]. And except the IκB expression [15, 19], the actors of the NF-κB pathways were never characterized. Having knowledge about the NF-κB subunits may have a functional relevance since the various homodimers and heterodimers display different DNA-binding and transactivation specificities [20–22]. No data about RelB or c-Rel activation was ever reported to date in TC. In this study, we provide the first in-depth characterization of the classical and alternative NF-κB pathways in ten PTC or ATC cell lines and the identification of the activated NF-κB dimers through their DNA-binding capacity in gel shift and supershift assays. This method is the most direct and informative technique since it offers the possibility to distinguish p50 binding in functional p50 heterodimers with RelA or RelB subunits from p50 binding in transactivation defective-p50/p50 homodimers. We thus demonstrated that the NF-κB activity was mainly supported by RelA/p50 dimers. Interestingly, we also provided the first demonstration of the activation of RelB in TC cell lines and PTCs samples. With the IKKα and p100 phosphorylation, that clearly evidences the activation of the alternative NF-κB pathway in thyroid cancer in addition to the classical one.

The huge number of NF-κB target genes [23] and the great variety of their cellular functions explain the extend of processes by which the NF-κB pathways are implicated in carcinogenesis [24, 25]. While an anti-apoptotic role of the constitutive NF-κB activity frequently contributes to the neoplastic properties, it was barely reported in TC [17, 19] or observed like a protective role against drug-induced apoptosis [26]. The diversity of the cellular models and of the experimental strategies used (pharmacological inhibitors with various specificity, inhibition by the super-repressor IκB mutant) probably explains the contradictory results about the proliferative role of the NF-κB pathway in TC [16, 17, 26–28]. By specifically inhibiting the classical and alternative NF-κB pathways through the RelA or RelB downregulation, our study shows that they are not or very slightly implicated in cell proliferation, except in the TTA1 cell line. The high level of the constitutive NF-κB activity in this cell line that we recently demonstrated induced by the c-MET overexpression [29] could explain its higher dependence on the NF-κB proteins.

The transcriptome meta-analysis is a powerful way to identify deregulated signaling pathways while also giving lighting of their pathological impact. Targeted on the differential expression of a set of NF-κB target genes, our analysis of the large TCGA PTC cohort clearly highlights the activation of the NF-κB signaling in PTC. Interestingly, our study identifies differential NF-κB related-gene expression programs in BRAF- or RAS-mutated PTCs. These differential NF-κB signatures can have biological consequences, as observed with increased MAPK signaling activation in BRAF-mutated PTCs, promoting greater thyroid dedifferentiation [12, 30]. Another key point of our study is the identification of a NF-κB signature indicating the involvement of NF-κB activity in tumoral progression specifically in BRAF-mutated PTC. That is in agreement with published data demonstrating a correlation between RelA nuclear localization and extrathyroid extension, metastatic lymph nodes and BRAF^V600E^ mutation [18, 31]. By using the RNA targeting strategy in a papillary (BCPAP) and an anaplastic (8505 C) cellular model, our study highlighted that the migration/invasive cell processes are activated by RelA, and also RelB, potentially through the expression of genes of the BRAF-specific NF-κB signatures (FN1, MMP1, PLAU, LCN2), some of them being under the control of RelA and/or RelB, as we demonstrated. Their implication in cell invasion and metastasis was frequently reported in various cancers [32–35], among them thyroid cancers [36–40]. Some studies also reported the NF-κB dependent cell migration and invasion of TC cell lines in association with MMPs expression [26, 41, 42]. Interestingly, some genes identified in our BRAF-specific NF-κB signature were recently described upregulated in radioactive iodine-refractory PTCs [43].

The mechanisms inducing constitutive NF-κB activation are largely unknown in TC. Given the contribution of the NF-κB signaling in TC tumor progression, identifying NF-κB upstream activators might prove to have some therapeutic interest for BRAF-advanced PTCs. We identified MAP3K14/NIK as an activator of the NF-κB pathways in TC since contributing to the NF-κB activity in BRAF-mutated cell lines. The NIK expression that we observed in PTC samples also indicated its implication in thyroid tumorigenesis. The NIK protein accumulation may result from a protein stabilization process since PTCs from the TCGA cohort didn’t display higher level of NIK mRNA compared to healthy tissue (data not shown). Already reported as implicated in the RET/PTC-induced NF-κB activity [44], NIK stabilization could be a common mechanism of NF-κB activation in PTC. NIK is the master regulator of the alternative NF-κB pathway [45], that is effectively activated in thyroid cancer as we demonstrated. We also showed that NIK expression is also required for the classical pathway activation (RelA/p50 dimers activation and IκB phosphorylation), as it was previously reported [46, 47]. Our study also shows that NIK downregulation impaired the cell invasion potential of PTC and ATC cell lines, evidencing NIK as a potential therapeutic target for progressive thyroid cancer. The recent data about its accumulation and implication in the growth and progression of many cancers (multiple myeloma, breast, pancreas, melanoma, kidney and colon) made NIK an attractive target for cancer therapy. NIK inhibitors with in vitro anti-tumor properties were recently developed but their pharmacokinetic properties need to be refined for in vivo evaluation [13]. Nevertheless, NIK targeting in cancers could benefit from NIK inhibitors validated in animal models of auto-immune and inflammatory diseases [48–50].

Improving the prognosis and treatment of advanced thyroid cancers also requires predictive biomarkers that would earlier identify patients at risk of metastatic disease who will need personalized targeting therapy. Several genomic, transcriptomic or proteomic studies provided increasing data about molecular features of metastatic progression of thyroid cancers [43, 51–53]. Our search for a NF-κB signature led to the identification of a transcriptomic signature of tumor progression in BRAF-mutated PTCs. A more extensive expression analysis in PTCs would be necessary to establish the predictive value for these NF-κB target genes as biomarkers for metastasis. A correlation of advanced stages of PTCs with the overexpression of FN1, PLAU, LCN2 and LGALS3 was already reported [36, 39, 54–56].

The efficiency of NF-κB targeting to reduce growth and chemoresistance in TC was demonstrated in some preclinical studies [57]. Another publication reported the limited benefice of combining NF-κB targeting with cytotoxic chemotherapy [58]. Likewise, the sole NF-κB targeting clinical trial (NCT00104871) with the proteasome inhibitor bortezomib didn’t show any therapeutic effect [59]. The development of NF-κB targeting strategy in advanced thyroid cancers requires a better understanding of the NF-κB pathway involvement in TC. This study presented a detailed analysis of the NF-κB signaling and its role in follicular-derived thyroid cancers. The activation of the alternative pathway together with the implication of the MAP3K NIK and their role in cell invasion process are novel findings that we expect opening new therapeutic perspectives for advanced thyroid cancers. In the same way, the identification of a BRAF-specific NF-κB signature for tumor progression will enable to determine biomarkers that aid in prognosis and in the evaluation of treatment efficacy.

## Materials and methods

### Cell lines

The human authentified ATC cell lines TTA1, ACT1, CAL62, C643, SW1736, HTh104 and the human authentified PTC cell lines TPC1 and KTC1 were kindly provided by J. Fagin (Memorial Sloan Kettering Cancer Center, New York, USA). The human ATC thyroid cell line 8505C and the human PTC cell line BCPAP were purchased from the German Collection of Microorganisms and Cell Culture. The SW1736, KTC1, HTh104, BCPAP and 8505C cell lines harbor the BRAF^T199A^ mutation, the ACT1, Cal62, C643 cell lines harbor the N-RAS^Q61K^, K-RAS^G12R^ or H-RAS^G13R^ mutation, respectively, the TPC1 cell line displays RET-PTC1 rearrangement [60] and the TTA1 cell line MET amplification [29]. The cell lines were tested for no mycoplasma contamination. The cell lines were cultured either in RPMI or in DMEM medium supplemented with 5-10% fetal calf sereum and antibiotics (Life Technologies).

### Patients

Samples of PTC tumors were collected at the Biological Resources Centers and Tumor Bank Platforms of Cochin Hospital (BB-0033-00023 certification) from patients referred as to the CAncer Research for Personalised Medicine (CARPEM) Institute upon written consent or non-opposition cohort (ethical approvals from National Ethical Committee CPP Ile-de-France).

### Reagents

The MEK inhibitors UO126 and selumetinib (AZD6244) were purchased from SelleckChemical LLC. The primary antibodies used are listed in table S5.

### siRNA transfection

Cells were transfected with 100 pmole siRNA with Lipofectamine RNAiMAX (Life Technologies) according to the manufacturer’s protocol. The siRNAs were purchased from Eurofins Genomics and are listed in table S6.

### Western blotting

Total cell protein extracts were prepared in NP40 lysis buffer as previously described [29]. Cytoplasmic and nuclear protein extracts were prepared by using the NE-PER nuclear and cytoplasmic extraction reagents (ThermoFisher Scientific). Protein concentration was determined using the Bradford reagent (BioRad). Equal amounts of proteins were electrophoresed and immunoblotted as previously described [29]. Immunostaining was revealed by chemiluminescence (Pierce^TM^ ECL or West Dura western blotting substrate, ThermoFisher Scientific) and vizualized by using the ImageQuant LAS4000 imaging system (GE Healthcare, Houston, USA).

### Electrophoretic mobility shift assay

Electrophoretic mobility shift assay (EMSA) was carried for analysis of NF-κB activation, as previously described [61]. Briefly, total cell extracts prepared in high salt buffer were analyzed by using the radiolabeled HIV-LTR tandem κB oligonucleotide as κB probe [62].

### RNA extraction and qRT-PCR

RNA extraction was performed with ReliaPrep^TM^ RNA Cell Miniprep System (Promega) according to manufacturer’s instructions. RNA were retrotranscripted in cDNA using the High-Capacity cDNA Reverse Transcription Kit (Applied Biosystems, ThermoFisher Scientific). Real-time PCR analysis was carried out with LightCycler 480 SYBR Green I Master (Roche) on Light Cycler 480 (Roche, Bâle, Switzerland). Relative mRNA expression was calculated using the ∆∆CT method and PPIA mRNA as endogenous control. Primer sequences are detailed in table S7.

### Cell viability assay

After transfection, cells were seeded in octuplicate in 96-well plates. Cell viability was assayed after 2 days in culture by using the crystal violet colorimetric assay as described by Hafliger and coll [63]. The absorbance of the solution was measured using a microplate spectrophotometer (Tecan, Männedord, Switzerlan) at a wavelenght of 595 nm.

### Cell migration and invasion assays

Cell migration and invasion were examined by Transwell polycarbonate membrane inserts (Corning) as previously described [64]. After overnight incubation at 37 °C, cells that invaded the underside of the membrane were stained with crystal violet. The total membrane was scanned and analyzed using ImageJ software.

### RNAseq data and gene expression analysis

Clinical and RNAseq data were downloaded from the TCGA portal (Oct-2014 and Sept-2021; https://portal.gdc.cancer.gov). We selected 392 samples: 286 PTCs harboring BRAF-T199A mutation, 48 PTCs harboring a RAS mutation and 58 healthy thyroid tissues. Raw data mapping and count were done on genome version GRCh38/hg38 according to TCGA Research Network (https://www.cancer.gov/tcga) analysis pipeline. We started analysis using read counts matrix for 392 samples and 60488 ensembl genes. All data analysis were performed using R (R Foundation for Statistical Computing, Vienna, Austria, https://www.R-project.org/) and RStudio software (RStudio Team, Boston, USA). Genes with read count mean through all samples lower than 10 were discarded for downstream analysis. Read count data were then linearized and quantile normalized using voom function from Limma R package [65]. To identify differentially expressed genes, we applied a one-way analysis of variance for MUTATION factor and made pairwise Tukey’s post hoc tests between groups. We then considered as significant genes with p-value < 0.01 and fold-change > 2 for upregulation and fold-change < -2 for downregulation.

The enrichment for gene sets was performed using GSEA version 4.1.0 software (Broad Institute of the Massachusetts Institute of Technology and Harvard; http://broad.mit.edu/gsea/) interrogating the “regulatory target gene sets” database and the “oncogenic gene sets” database from the MSigDB v7.5 geneset database [66]. Normalized enrichment scores (NES) reflect a statistically significant enrichment for *P* values < 0.05 and/or FDR (false discovery rate) values < 0.25 [67].

Differentialy expressed genes were analyzed using Ingenuity Pathway Analysis (IPA) software (Ingenuity Systems, www.ingenuity.com). The upstream regulators function provides the prediction of the activation of transcription factors and regulative molecules. The expression profile of a set of 363 validated NF-κB target genes (table S1) established from data from the site of the Gilmore’lab (https://www.bu.edu/nf-kb/the-gilmore-lab/) and the “http://bioinfo.lifl.fr/NF-KB/ “site was analyzed.

### Immunohistochemistry

Immunohistochemical staining was performed on formalin-fixed, paraffin-embedded PTC samples. Sections were deparaffinized, rehydrated, subjected to epitope antigen retrieval by boiling in citric acid buffer (0.01 mM, pH 6.0) and permeabilized with 0.2% Triton X100. Endogenous peroxydases were inactivated by incubation in 3% hydrogen peroxyde, and the non-specific antibody-binding sites were blocked in 10% normal horse serum. Primary monoclonal anti-NIK, anti-RelA or anti-RelB antibodies (Santa-Cruz) were incubated overnight at 4 °C, followed by incubation with the biotin-conjugated secondary anti-mouse or anti-rabbit Ig antibody. Samples were then treated with horseradish peroxydase (HRP)-streptavidin complex and stained for peroxydase activity with HRP substrate (DAB solution, Dako). Samples were then counterstained in hematoxylin, rinsed, dehydrated and mounted. Whole slide images were digitized at 40x using the HPF-NanoZoomer RS 2.0 slide scanner (Hamamatsu, Hamamatsu-City, Japan) or the Lamina slide scanner (Akoya Perkin Elmer, USA) and images were collected using the NDP.view (Hamamatsu) or CaseViewer (3DHISTECH, Budapest,Hungary) softwares.

### Statistical analysis

Statistical analysis were performed by using GraphPad software on line. The data are expressed as mean ± Standard Deviation (SD) of 3 experiments unless otherwise specified. Statistical significance was assessed using the Student’s *t*-test (GraphPad software on line). *P* values < 0.05 were considered significant with the following degrees: * *p* < 0,05; ** *p* < 0,01; *** *p* < 0,001.

### Supplementary information

Supplementary informations

Supplemental table 1

Supplemental table 2

Supplemental table 3

## Data Availability

Data are available from the corresponding author on reasonable request.
