# Supplementary material for: NF-κB signaling activation and roles in thyroid cancers: implication of MAP3K14/NIK
Source: Oncogenesis. 2023 Nov 16;12(1):55. doi: 10.1038/s41389-023-00496-w (PMC10654696; doi:10.1038/s41389-023-00496-w)
Supplement: Supplementary file 1 — Supplementary informations [file 41389_2023_496_MOESM1_ESM.pdf]

Supplementary informations

NF-κB signaling activation and roles in thyroid cancers: implication of MAP3K14/NIK

Supplemental figures.

Fig. S1. The downregulation of RelA or RelB mRNA in the viability, migration and invasion assays reported in Fig.2.

Fig. S2. The NF-κB related gene expression distinguishes BRAF- and RAS-mutated PTCs.

Fig. S3. The MAPK pathway is not involved in the constitutive activation of NF-κB pathways in BRAF-mutated cell lines.

Fig. S4. The downregulation of NIK mRNA in the EMSA, immunoblot and migration/invasion assays.

Fig. S5. Representative images showing cytoplasmic and nuclear localization of RelA in PTC samples.

Supplemental tables

Table S4. Clinical characteristics of patients with PTC.

Table S5. Primary antibodies used for immunoblotting and supershift experiments.

Table S6. List of siRNA used for RNA-targeting.

Table S7. Primers sequences used for qRT-PCR.

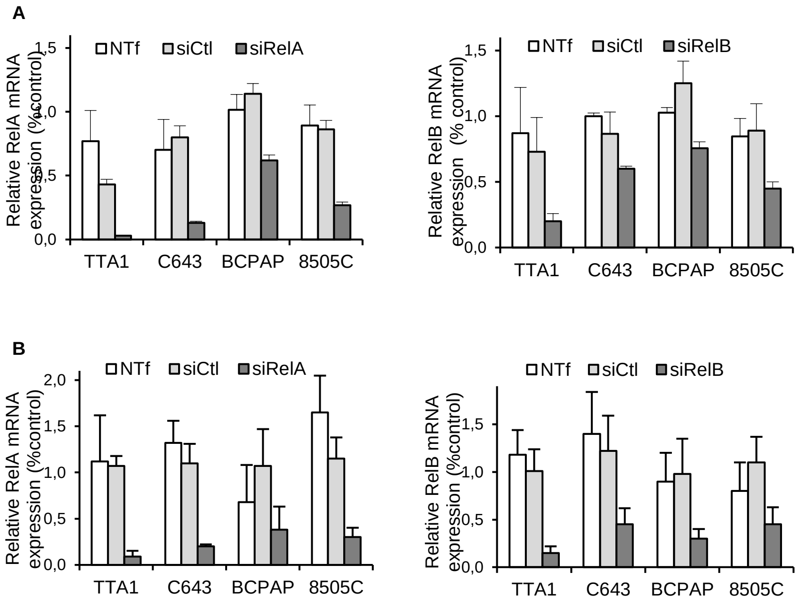

Fig. S1. The downregulation of RelA or RelB mRNA in the viability, migration and invasion assays reported in Fig.2. RelA and RelB mRNA expression analysis by qRT-PCR in transfected cell lines used for viability (A), and cell migration and invasion assays (B).

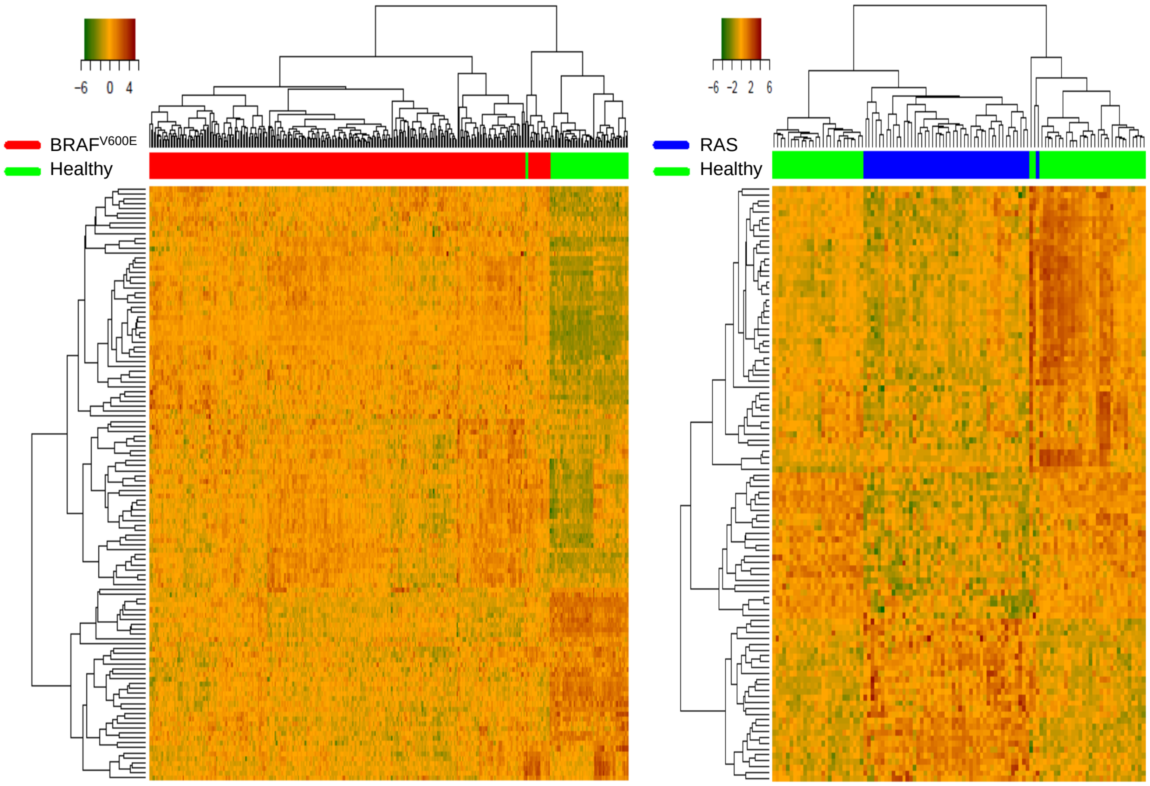

Fig. S2. The NF-κB related gene expression distinguishes BRAF- and RAS-mutated PTCs. Heatmaps of the unsupervised clustering of BRAF-mutated PTCs (N=286; left panel) or RAS-mutated PTCs (N=48; right panel) and healthy thyroid tissues (N=58). The clustering of BRAF-PTCs is based on the mRNA expression of the 121 differentially expressed NF-κB target genes (fc>2; p<0.01) in BRAF-mutated PTCs and that of RAS-mutated PTCs is based on the mRNA expression of the 102 differentially expressed NF-κB target genes (fc>2; p<0.01) in RAS-mutated PTCs.

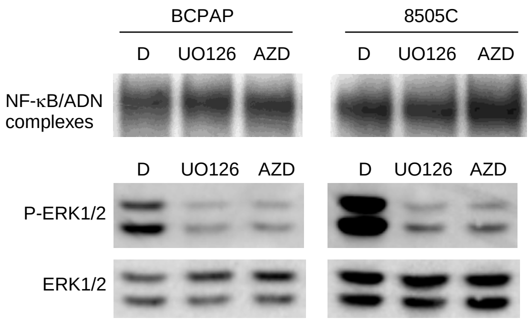

Fig. S3. The MAPK pathway is not involved in the constitutive activation of NF-κB pathways in BRAF-mutated cell lines. BCPAP and 8505C cells were incubated with UO126, AZD6244 (AZD) or DMSO (D) as negative control for 24h. NF-κB activity was analyzed in EMSA experiments (upper panels), and p-ERK and total ERK expression was analyzed in immunoblots (lower panels).

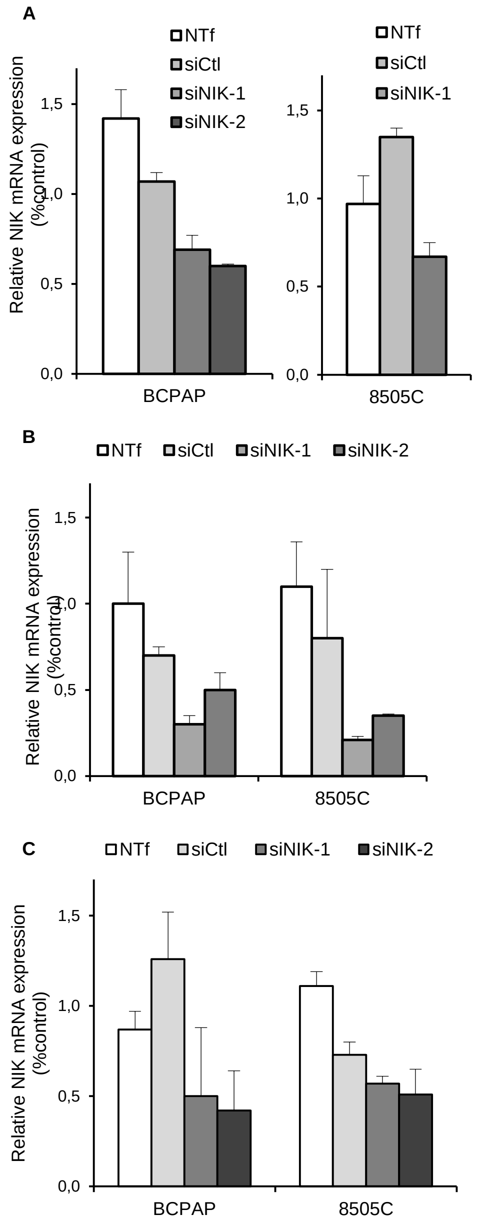

Fig. S4. The downregulation of NIK mRNA in the EMSA, immunoblot and migration/invasion assays. NIK mRNA expression analysis by qRT-PCR in BCPAP and 8505C transfected cells used for EMSA experiments reported in Fig. 6A (**A**), for immunoblot analysis reported in Fig. 6B **(B)** and for cell migration and invasion assays reported in Fig.6C (**C**).

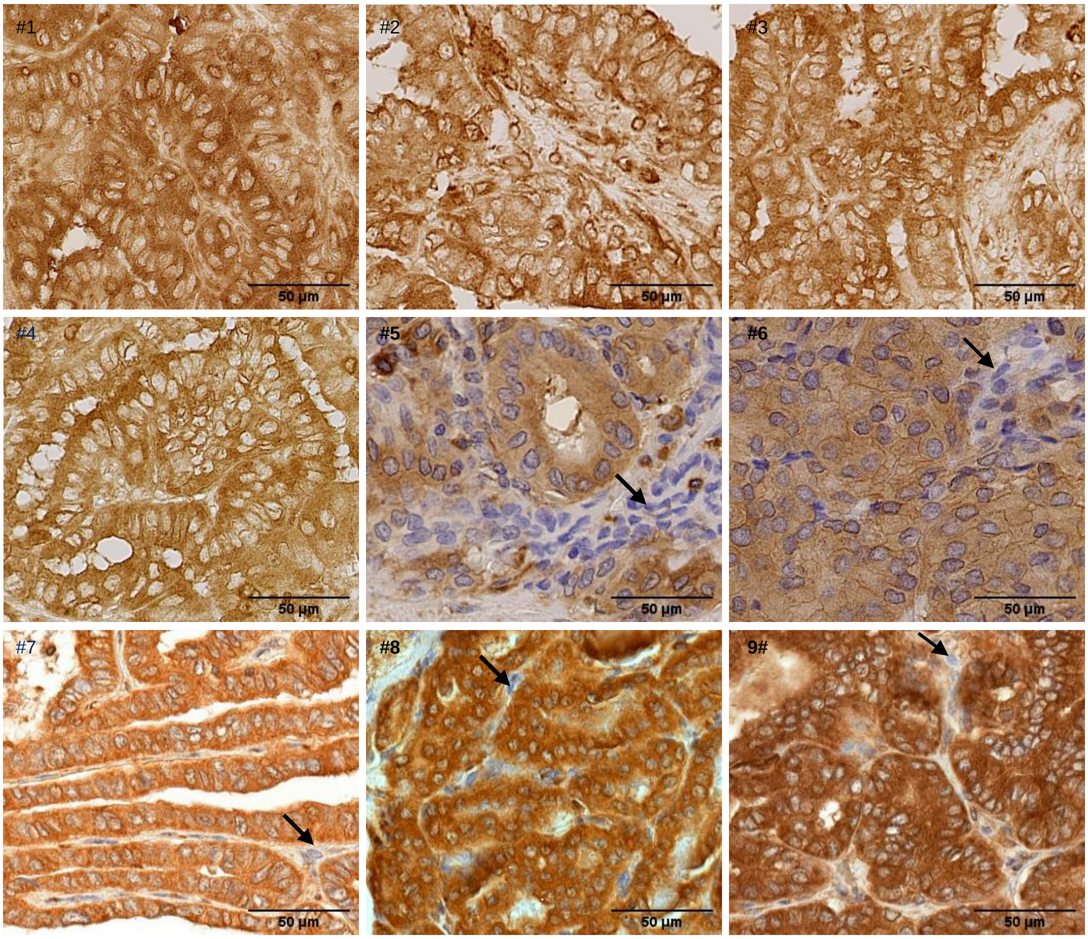

Fig. S5. Representative images showing cytoplasmic and nuclear localization of RelA in PTC samples. Paraffin-embedded sections of 9 PTC samples were immunostained using anti-RelA antibody (samples #1, #2, #3 and #4) or immunostained using anti-RelA antibody and counterstained with hematoxylin (samples #5, #6, #7, #8 and #9). Nuclear immunoreactivity is indicated by a slight mauve staining of nuclei in samples #5, #6, #7, #8 and #9, compared to nuclei of stromal cells (indicated by arrows) which displayed blue staining. No hematoxylin staining in samples #1, #2, #3 and #4 allowed to detected RelA immunopositivity in nuclei of tumoral cells with a slight peroxydase-positive staining.

Supplemental table 4: Clinical characteristics of patients with PTC

Patient N°	Type	Sex	Age	Tumor size (cm)	TNM *	Invasion	Extension	Lymph nodes	Risk$
1	classical	F	51	1.3	pT1b N0	+	-	0/6	low
2	encapsulated §	M	24	1.8	pT1b N1a	+	-	8/14	int
3	encapsulated §	F	36	1.2	pT1b Nx	minimal	-	NA	int
4	classical	F	23	1.5	pT2 N1a	+	-	2/9	int
5	classical	F	45	2.5	pT3b N1a	+	+	3/17	high
6	solid/trabecular	M	59	6.7	pT3b Nx	+	+	NA	high
7	classical	F	40	2	pT2 N0	+	-	0/5	int
8	oncocytic	M	69	1.4	pT3 Nx	-	+	NA	high
9	classical	F	55	0.9	pT1 N0	-	-	0/0	low

*: pathological TNM staging
$: ATA (American Thyroid Association) risk stratification
§ : invasive encapsulated follicular variant
int: intermediate

Supplemental table 5 : Primary antibodies used for immunoblotting and supershift experiments

Antibody target	Phosphorylation site, if applicable	Host	Supplier / Catalog#
IκB, alpha		rabbit	Santa Cruz / sc-847
phospho-IκB,alpha	Ser32/36	mouse	Cell Signaling / 9246
IKK, alpha		rabbit	Santa Cruz / sc-7182
IKK, beta		rabbit	Cell Signaling / 2678
phospho-IKK	Ser176/180	rabbit	Cell Signaling / 2697
NF-κB1/p105		rabbit	Cell Signaling / 3586
phospho-NF-κB1	Ser933	rabbit	Cell Signaling / 4806
NF-κB2/p100		rabbit	Cell Signaling / 4882
NF-κB2/p100		rabbit	Santa Cruz / sc-114X
phospho-NF-κB2	Ser866/870	rabbit	Cell Signaling / 4810
NIK		rabbit	Cell Signaling / 4994
NIK		mouse	Santa Cruz / sc-8417
RelA / p65		rabbit	Santa Cruz / sc-372 / sc-372X
RelB		rabbit	Santa Cruz / sc-226 / sc-226X
c-Rel		rabbit	Santa Cruz / sc-70X
HDAC		mouse	Cell Signaling / 5356
GAPDH		rabbit	Santa Cruz / sc-25778

Supplemental table 6 : List of siRNA used for RNA-targeting.

Gene target	Sequence 5' to 3'
Ctl 1	5'-AAAUGGGUGGAGCUCUUGA-3'
Ctl 2	5'-CAGUCGCGUUUGCGACUGG-3'
Ctl 3	5'-GCGAGCAACCGAACCUAAA-3'
RelA	5'-GGAUUGAGGAGAAACGUAA-3'
RelB	5'-GACTGCACCGACGGCATCT-3'
NIK 1	5'-UACCUCCACUCACGAAGGA-3'
NIK 2	5'-GCCAGTCCGAGAGTCTTGATCAGAT-3'

Supplemental table 7 : Primers sequences used for qRT-PCR.

Target gene	Forward primer (5'>3')	Reverse primer (5'>3')	Prod. size (bp)
CCL20	CTGGCTGCTTTGATGTCAGT	CGTGTGAAGCCCACAATAAA	128
LGALS3	CTTATAACCTGCCTTTGCCTGG	GCAACATCATTCCCTCTTTGGA	118
LCN2	GAAGACAAAGACCCGCAAAAG	CTGGCAACCTGGAACAAAAG	135
MMP1	CAGATTCTACATGCGCACAAAT	CTTTGAAAAACCGGACTTCATC	137
NIK	CCAGCTGCCATCTCTATCATC	AAAGGTGGGGCTGAACTCTT	66
PPIA	ATGGCACTGGTGGCAAGTCC	TTGCCATTCCTGGACCCAAA	241
PLAU	CCAAAGGCAGCAATGAACT	CCCCTCATAGCAGGTTTTTG	167
RelA	TTGAGCCCACAAAGCCTTATCAAGT	GGACAATGCCAGTGCCATACAG	104
RelB	CTCACTCTCGCTCGCCGTTTC	CACAGGGCCCAGGGTGACCGT	172
