# Supplementary material for: NF-κB signaling activation and roles in thyroid cancers: implication of MAP3K14/NIK
Source: Oncogenesis. 2023 Nov 16;12(1):55. doi: 10.1038/s41389-023-00496-w (PMC10654696; doi:10.1038/s41389-023-00496-w)
Supplement: Supplementary file 3 — Supplemental table 2 [file 41389_2023_496_MOESM3_ESM.doc]

**Supplemental table 2: List of the significantly deregulated genes from the set of 363 validated NF-κB target genes in BRAF-mutated PTCs compared to healthy thyroid tissues.** (from Ingenuity Pathway Analysis with p-value < 0.01 and fold change >2 parameters).

| **Symbol** | **Entrez Gene Name** | **Expr p-value** | **Expr Fold Change** | **Location** |
| --- | --- | --- | --- | --- |
| FN1 | fibronectin 1 | 5,28E-128 | 75,316 | Extracellular Space |
| CSF2 | colony stimulating factor 2 | 4,98E-119 | 62,08 | Extracellular Space |
| KRT15 | keratin 15 | 7,36E-115 | 49,841 | Cytoplasm |
| CHI3L1 | chitinase 3 like 1 | 8,31E-58 | 39,441 | Extracellular Space |
| SERPINA1 | serpin family A member 1 | 5,11E-161 | 38,981 | Extracellular Space |
| LCN2 | lipocalin 2 | 5,59E-56 | 38,034 | Extracellular Space |
| CXCL5 | C-X-C motif chemokine ligand 5 | 3,97E-30 | 14,225 | Extracellular Space |
| CCL20 | C-C motif chemokine ligand 20 | 7,49E-37 | 12,851 | Extracellular Space |
| MMP1 | matrix metallopeptidase 1 | 6,55E-39 | 12,575 | Extracellular Space |
| ALOX5 | arachidonate 5-lipoxygenase | 7,87E-39 | 12,364 | Cytoplasm |
| PLAU | plasminogen activator, urokinase | 1,22E-56 | 10,471 | Extracellular Space |
| CCL17 | C-C motif chemokine ligand 17 | 1,17E-24 | 10,378 | Extracellular Space |
| KISS1 | KiSS-1 metastasis suppressor | 4,70E-35 | 8,735 | Cytoplasm |
| LGALS3 | galectin 3 | 4,37E-136 | 8,1 | Extracellular Space |
| ADORA1 | adenosine A1 receptor | 3,63E-88 | 7,889 | Plasma Membrane |
| MDK | midkine | 3,02E-68 | 7,461 | Extracellular Space |
| EBI3 | Epstein-Barr virus induced 3 | 8,61E-50 | 7,417 | Extracellular Space |
| SDC4 | syndecan 4 | 1,27E-118 | 7,192 | Plasma Membrane |
| TNC | tenascin C | 2,95E-34 | 6,754 | Extracellular Space |
| ICAM1 | intercellular adhesion molecule 1 | 1,04E-74 | 6,343 | Plasma Membrane |
| ELF3 | E74 like ETS transcription factor 3 | 3,37E-64 | 6,295 | Nucleus |
| UPP1 | uridine phosphorylase 1 | 1,05E-74 | 5,739 | Cytoplasm |
| DMP1 | dentin matrix acidic phosphoprotein 1 | 6,16E-15 | 5,375 | Extracellular Space |
| APOE | apolipoprotein E | 2,50E-19 | 5,283 | Extracellular Space |
| TGM1 | transglutaminase 1 | 2,93E-49 | 4,756 | Plasma Membrane |
| CXCL8 | C-X-C motif chemokine ligand 8 | 5,80E-20 | 4,668 | Extracellular Space |
| FSTL3 | follistatin like 3 | 5,67E-69 | 4,372 | Extracellular Space |
| CCL22 | C-C motif chemokine ligand 22 | 1,30E-15 | 4,258 | Extracellular Space |
| TNFSF15 | TNF superfamily member 15 | 9,18E-46 | 4,235 | Extracellular Space |
| S100A6 | S100 calcium binding protein A6 | 5,26E-67 | 4,136 | Cytoplasm |
| CFB | complement factor B | 1,54E-21 | 4,106 | Extracellular Space |
| GRIN1 | glutamate ionotropic receptor NMDA type subunit 1 | 3,94E-20 | 4,059 | Plasma Membrane |
| TGM2 | transglutaminase 2 | 9,99E-32 | 4,026 | Cytoplasm |
| IL1RN | interleukin 1 receptor antagonist | 1,05E-24 | 3,978 | Extracellular Space |
| KRT6B | keratin 6B | 5,40E-09 | 3,948 | Cytoplasm |
| SAA2 | serum amyloid A2 | 3,08E-08 | 3,846 | Extracellular Space |
| SPP1 | secreted phosphoprotein 1 | 6,57E-16 | 3,797 | Extracellular Space |
| OLR1 | oxidized low density lipoprotein receptor 1 | 9,22E-19 | 3,761 | Plasma Membrane |
| PRDM1 | PRSET domain 1 | 3,09E-42 | 3,631 | Nucleus |
| CCND1 | cyclin D1 | 5,67E-73 | 3,587 | Nucleus |
| SAA1 | serum amyloid A1 | 1,76E-07 | 3,468 | Extracellular Space |
| OXTR | oxytocin receptor | 1,10E-48 | 3,445 | Plasma Membrane |
| MMP3 | matrix metallopeptidase 3 | 3,65E-07 | 3,301 | Extracellular Space |
| IL11 | interleukin 11 | 1,92E-13 | 3,175 | Extracellular Space |
| C3 | complement C3 | 3,26E-15 | 3,027 | Extracellular Space |
| S100A4 | S100 calcium binding protein A4 | 2,44E-19 | 2,944 | Cytoplasm |
| MMP9 | matrix metallopeptidase 9 | 1,21E-07 | 2,772 | Extracellular Space |
| PLK3 | polo like kinase 3 | 1,20E-43 | 2,752 | Nucleus |
| IGFBP2 | insulin like growth factor binding protein 2 | 8,07E-24 | 2,698 | Extracellular Space |
| CX3CL1 | C-X3-C motif chemokine ligand 1 | 2,60E-39 | 2,665 | Extracellular Space |
| XDH | xanthine dehydrogenase | 9,98E-06 | 2,609 | Cytoplasm |
| CXCL1 | C-X-C motif chemokine ligand 1 | 8,39E-08 | 2,531 | Extracellular Space |
| NOD2 | nucleotide binding oligomerization domain containing 2 | 4,74E-15 | 2,476 | Cytoplasm |
| C4A/C4B | complement C4A (Rodgers blood group) | 1,88E-11 | 2,459 | Extracellular Space |
| CCND2 | cyclin D2 | 3,17E-50 | 2,452 | Nucleus |
| BCL2L1 | BCL2 like 1 | 2,69E-51 | 2,428 | Cytoplasm |
| IL23A | interleukin 23 subunit alpha | 1,78E-31 | 2,414 | Extracellular Space |
| PGR | progesterone receptor | 9,88E-13 | 2,409 | Nucleus |
| SH3BGRL3 | SH3 domain binding glutamate rich protein like 3 | 1,61E-38 | 2,407 | Nucleus |
| HMOX1 | heme oxygenase 1 | 2,96E-17 | 2,397 | Cytoplasm |
| FAS | Fas cell surface death receptor | 2,03E-29 | 2,392 | Plasma Membrane |
| PYCARD | PYD and CARD domain containing | 1,51E-22 | 2,376 | Cytoplasm |
| DNASE1L2 | deoxyribonuclease 1 like 2 | 2,04E-11 | 2,337 | Extracellular Space |
| NOS2 | nitric oxide synthase 2 | 6,12E-11 | 2,336 | Cytoplasm |
| CXCL2 | C-X-C motif chemokine ligand 2 | 7,16E-07 | 2,336 | Extracellular Space |
| LYZ | lysozyme | 5,79E-07 | 2,313 | Extracellular Space |
| BAX | BCL2 associated X, apoptosis regulator | 2,84E-44 | 2,289 | Cytoplasm |
| INHBA | inhibin subunit beta A | 4,11E-08 | 2,236 | Extracellular Space |
| NOX1 | NADPH oxidase 1 | 3,65E-31 | 2,211 | Cytoplasm |
| CD44 | CD44 molecule (Indian blood group) | 8,69E-38 | 2,205 | Plasma Membrane |
| KCNK5 | potassium two pore domain channel subfamily K member 5 | 1,14E-46 | 2,186 | Plasma Membrane |
| SPI1 | Spi-1 proto-oncogene | 5,66E-13 | 2,165 | Nucleus |
| AGT | angiotensinogen | 7,31E-09 | 2,145 | Extracellular Space |
| BRCA2 | BRCA2 DNA repair associated | 1,05E-23 | 2,091 | Nucleus |
| KCNN2 | potassium calcium-activated channel subfamily N member 2 | 6,40E-16 | 2,078 | Plasma Membrane |
| APOBEC2 | apolipoprotein B mRNA editing enzyme catalytic subunit 2 | 2,34E-09 | 2,076 | Other |
| CCL23 | C-C motif chemokine ligand 23 | 2,24E-07 | 2,062 | Extracellular Space |
| LTB | lymphotoxin beta | 1,69E-04 | 2,055 | Extracellular Space |
| PLCD1 | phospholipase C delta 1 | 9,94E-37 | 2,047 | Cytoplasm |
| BLNK | B cell linker | 5,88E-20 | 2,046 | Cytoplasm |
| THBS1 | thrombospondin 1 | 1,04E-06 | 2,037 | Extracellular Space |
| CALCB | calcitonin related polypeptide beta | 1,95E-03 | 2,036 | Extracellular Space |
| BCL2L11 | BCL2 like 11 | 1,89E-45 | -2,033 | Cytoplasm |
| HGF | hepatocyte growth factor | 3,47E-12 | -2,055 | Extracellular Space |
| CD40LG | CD40 ligand | 1,39E-06 | -2,083 | Extracellular Space |
| CYP27B1 | cytochrome P450 family 27 subfamily B member 1 | 1,40E-22 | -2,125 | Cytoplasm |
| PAX8 | paired box 8 | 2,18E-44 | -2,13 | Nucleus |
| ASPH | aspartate beta-hydroxylase | 3,61E-38 | -2,158 | Cytoplasm |
| NAIP | NLR family apoptosis inhibitory protein | 2,83E-15 | -2,204 | Cytoplasm |
| PTGDS | prostaglandin D2 synthase | 6,07E-06 | -2,281 | Cytoplasm |
| GADD45B | growth arrest and DNA damage inducible beta | 7,54E-14 | -2,318 | Cytoplasm |
| IL12A | interleukin 12A | 1,34E-15 | -2,389 | Extracellular Space |
| ST8SIA1 | ST8 alpha-N-acetyl-neuraminide alpha-2,8-sialyltransferase 1 | 1,12E-11 | -2,408 | Cytoplasm |
| CXCR5 | C-X-C motif chemokine receptor 5 | 5,82E-06 | -2,418 | Plasma Membrane |
| ERVW-1 | endogenous retrovirus group W member 1, envelope | 1,19E-09 | -2,487 | Plasma Membrane |
| ST6GAL1 | ST6 beta-galactoside alpha-2,6-sialyltransferase 1 | 9,90E-18 | -2,516 | Cytoplasm |
| GBP1 | guanylate binding protein 1 | 4,05E-17 | -2,52 | Cytoplasm |
| PTX3 | pentraxin 3 | 1,77E-27 | -2,595 | Extracellular Space |
| CSF3 | colony stimulating factor 3 | 2,39E-04 | -2,807 | Extracellular Space |
| POMC | proopiomelanocortin | 1,11E-13 | -2,844 | Extracellular Space |
| NUAK2 | NUAK family kinase 2 | 3,62E-53 | -2,847 | Nucleus |
| SNAI1 | snail family transcriptional repressor 1 | 9,59E-29 | -3,061 | Nucleus |
| DUSP1 | dual specificity phosphatase 1 | 2,41E-20 | -3,246 | Nucleus |
| CCL19 | C-C motif chemokine ligand 19 | 5,71E-05 | -3,329 | Extracellular Space |
| LIPG | lipase G, endothelial type | 1,93E-28 | -3,46 | Extracellular Space |
| AICDA | activation induced cytidine deaminase | 6,99E-10 | -3,787 | Cytoplasm |
| BMP2 | bone morphogenetic protein 2 | 8,76E-33 | -3,841 | Extracellular Space |
| CYP7B1 | cytochrome P450 family 7 subfamily B member 1 | 1,27E-47 | -3,891 | Cytoplasm |
| CR2 | complement C3d receptor 2 | 4,84E-08 | -4,422 | Plasma Membrane |
| HSD11B2 | hydroxysteroid 11-beta dehydrogenase 2 | 1,76E-34 | -4,732 | Cytoplasm |
| BCL2 | BCL2 apoptosis regulator | 1,08E-78 | -4,886 | Cytoplasm |
| AR | androgen receptor | 7,26E-47 | -5,004 | Nucleus |
| APOD | apolipoprotein D | 6,35E-26 | -5,257 | Extracellular Space |
| RAG1 | recombination activating 1 | 5,86E-61 | -5,747 | Nucleus |
| DIO2 | iodothyronine deiodinase 2 | 2,12E-53 | -6,443 | Cytoplasm |
| AQP4 | aquaporin 4 | 9,42E-29 | -7,731 | Plasma Membrane |
| KLK3 | kallikrein related peptidase 3 | 3,29E-13 | -8,428 | Extracellular Space |
| NOS1 | nitric oxide synthase 1 | 3,73E-39 | -9,027 | Cytoplasm |
| NCAM1 | neural cell adhesion molecule 1 | 2,98E-38 | -11,542 | Plasma Membrane |
| RAG2 | recombination activating 2 | 3,24E-75 | -136,707 | Nucleus |
| TFF3 | trefoil factor 3 | 3,95E-70 | -162,441 | Extracellular Space |
