# Supplementary material for: NF-κB signaling activation and roles in thyroid cancers: implication of MAP3K14/NIK
Source: Oncogenesis. 2023 Nov 16;12(1):55. doi: 10.1038/s41389-023-00496-w (PMC10654696; doi:10.1038/s41389-023-00496-w)
Supplement: Supplementary file 4 — Supplemental table 3 [file 41389_2023_496_MOESM4_ESM.doc]

**Supplemental table 3: List of the significantly deregulated genes from the set of 363 validated NF-κB target genes in RAS-mutated PTCs compared to healthy thyroid tissues**. (from Ingenuity Pathway Analysis with p-value < 0.01 and fold change >2 parameters).

| **Symbol** | **Entrez Gene Name** | **Expr p-value** | **Expr Fold Change** | **Location** |
| --- | --- | --- | --- | --- |
| CSF2 | colony stimulating factor 2 | 2,02E-15 | 6,075 | Extracellular Space |
| KRT15 | keratin 15 | 1,28E-10 | 5,174 | Cytoplasm |
| MMP1 | matrix metallopeptidase 1 | 1,62E-07 | 5,084 | Extracellular Space |
| NQO1 | NAD(P)H quinone dehydrogenase 1 | 2,22E-18 | 4,387 | Cytoplasm |
| GRIN1 | glutamate ionotropic receptor NMDA type subunit 1 | 7,47E-11 | 4,237 | Plasma Membrane |
| APOBEC2 | apolipoprotein B mRNA editing enzyme catalytic subunit 2 | 5,62E-14 | 4 | Other |
| ORM1 | orosomucoid 1 | 1,14E-08 | 3,973 | Extracellular Space |
| ADORA1 | adenosine A1 receptor | 6,37E-22 | 3,87 | Plasma Membrane |
| KISS1 | KiSS-1 metastasis suppressor | 2,25E-13 | 3,864 | Cytoplasm |
| LCN2 | lipocalin 2 | 2,21E-06 | 3,559 | Extracellular Space |
| DNASE1L2 | deoxyribonuclease 1 like 2 | 2,99E-16 | 3,267 | Extracellular Space |
| CCND1 | cyclin D1 | 7,48E-32 | 3,224 | Nucleus |
| PRDM1 | PRSET domain 1 | 1,52E-14 | 3,106 | Nucleus |
| CD44 | CD44 molecule (Indian blood group) | 5,66E-23 | 2,821 | Plasma Membrane |
| ENG | endoglin | 1,71E-16 | 2,716 | Plasma Membrane |
| ABCC6 | ATP binding cassette subfamily C member 6 | 2,25E-29 | 2,622 | Plasma Membrane |
| CCND2 | cyclin D2 | 2,86E-20 | 2,58 | Nucleus |
| DMP1 | dentin matrix acidic phosphoprotein 1 | 1,80E-06 | 2,566 | Extracellular Space |
| KCNN2 | potassium calcium-activated channel subfamily N member 2 | 1,29E-12 | 2,559 | Plasma Membrane |
| KRT6B | keratin 6B | 2,19E-06 | 2,465 | Cytoplasm |
| PDGFB | platelet derived growth factor subunit B | 4,77E-22 | 2,409 | Extracellular Space |
| TNFSF15 | TNF superfamily member 15 | 1,20E-09 | 2,406 | Extracellular Space |
| IGFBP2 | insulin like growth factor binding protein 2 | 1,96E-14 | 2,219 | Extracellular Space |
| KCNK5 | potassium two pore domain channel subfamily K member 5 | 3,93E-16 | 2,213 | Plasma Membrane |
| MDK | midkine | 3,32E-07 | 2,166 | Extracellular Space |
| BAX | BCL2 associated X, apoptosis regulator | 9,60E-16 | 2,129 | Cytoplasm |
| UPP1 | uridine phosphorylase 1 | 2,50E-10 | 2,121 | Cytoplasm |
| CTSB | cathepsin B | 9,10E-14 | 2,061 | Cytoplasm |
| CD274 | CD274 molecule | 6,85E-08 | -2,006 | Plasma Membrane |
| CCL23 | C-C motif chemokine ligand 23 | 5,13E-05 | -2,025 | Extracellular Space |
| IL1A | interleukin 1 alpha | 4,45E-04 | -2,041 | Extracellular Space |
| PIM1 | Pim-1 proto-oncogene, serinethreonine kinase | 3,64E-09 | -2,057 | Cytoplasm |
| PRF1 | perforin 1 | 1,16E-06 | -2,089 | Cytoplasm |
| AGER | advanced glycosylation end-product specific receptor | 5,01E-06 | -2,122 | Plasma Membrane |
| MYC | MYC proto-oncogene, bHLH transcription factor | 5,28E-07 | -2,167 | Nucleus |
| BMP4 | bone morphogenetic protein 4 | 1,04E-10 | -2,173 | Extracellular Space |
| RAG1 | recombination activating 1 | 9,33E-07 | -2,186 | Nucleus |
| CXCL2 | C-X-C motif chemokine ligand 2 | 1,05E-03 | -2,193 | Extracellular Space |
| C3 | complement C3 | 6,97E-05 | -2,209 | Extracellular Space |
| LYZ | lysozyme | 3,60E-03 | -2,242 | Extracellular Space |
| DUSP1 | dual specificity phosphatase 1 | 2,92E-06 | -2,298 | Nucleus |
| FASLG | Fas ligand | 4,59E-06 | -2,347 | Extracellular Space |
| TNFAIP3 | TNF alpha induced protein 3 | 1,37E-09 | -2,383 | Nucleus |
| POMC | proopiomelanocortin | 5,31E-05 | -2,443 | Extracellular Space |
| PTGS2 | prostaglandin-endoperoxide synthase 2 | 6,63E-06 | -2,463 | Cytoplasm |
| TFPI2 | tissue factor pathway inhibitor 2 | 7,30E-09 | -2,465 | Extracellular Space |
| VCAM1 | vascular cell adhesion molecule 1 | 5,16E-07 | -2,468 | Plasma Membrane |
| IL2RA | interleukin 2 receptor subunit alpha | 8,05E-07 | -2,472 | Plasma Membrane |
| THBS2 | thrombospondin 2 | 4,98E-10 | -2,499 | Extracellular Space |
| CXCL3 | C-X-C motif chemokine ligand 3 | 2,02E-05 | -2,54 | Extracellular Space |
| BCL2L11 | BCL2 like 11 | 5,75E-28 | -2,547 | Cytoplasm |
| NUAK2 | NUAK family kinase 2 | 4,74E-15 | -2,554 | Nucleus |
| NPY1R | neuropeptide Y receptor Y1 | 5,93E-11 | -2,572 | Plasma Membrane |
| IL12A | interleukin 12A | 5,75E-13 | -2,574 | Extracellular Space |
| NCAM1 | neural cell adhesion molecule 1 | 2,97E-10 | -2,597 | Plasma Membrane |
| HAS1 | hyaluronan synthase 1 | 7,90E-04 | -2,603 | Plasma Membrane |
| CCL22 | C-C motif chemokine ligand 22 | 7,83E-04 | -2,607 | Extracellular Space |
| GBP1 | guanylate binding protein 1 | 9,97E-06 | -2,612 | Cytoplasm |
| HSD11B2 | hydroxysteroid 11-beta dehydrogenase 2 | 5,93E-11 | -2,614 | Cytoplasm |
| CCR5 | C-C motif chemokine receptor 5 | 1,32E-06 | -2,641 | Plasma Membrane |
| IL32 | interleukin 32 | 7,55E-08 | -2,651 | Extracellular Space |
| PTX3 | pentraxin 3 | 3,26E-17 | -2,686 | Extracellular Space |
| SOX9 | SRY-box transcription factor 9 | 5,63E-19 | -2,699 | Nucleus |
| TNFSF13B | TNF superfamily member 13b | 1,55E-10 | -2,714 | Extracellular Space |
| GATA3 | GATA binding protein 3 | 7,47E-08 | -2,743 | Nucleus |
| CCL4 | C-C motif chemokine ligand 4 | 7,61E-06 | -2,758 | Extracellular Space |
| LTA | lymphotoxin alpha | 2,32E-04 | -2,844 | Extracellular Space |
| CR2 | complement C3d receptor 2 | 7,25E-03 | -2,942 | Plasma Membrane |
| CXCL10 | C-X-C motif chemokine ligand 10 | 2,84E-04 | -3,066 | Extracellular Space |
| CFB | complement factor B | 1,90E-06 | -3,106 | Extracellular Space |
| BMP2 | bone morphogenetic protein 2 | 6,20E-10 | -3,12 | Extracellular Space |
| TNFRSF9 | TNF receptor superfamily member 9 | 1,21E-04 | -3,209 | Plasma Membrane |
| CCR7 | C-C motif chemokine receptor 7 | 6,77E-06 | -3,266 | Plasma Membrane |
| LTB | lymphotoxin beta | 2,17E-04 | -3,316 | Extracellular Space |
| HGF | hepatocyte growth factor | 7,65E-16 | -3,443 | Extracellular Space |
| TNF | tumor necrosis factor | 1,06E-08 | -3,5 | Extracellular Space |
| CD48 | CD48 molecule | 1,79E-07 | -3,532 | Plasma Membrane |
| CCL5 | C-C motif chemokine ligand 5 | 7,09E-09 | -3,588 | Extracellular Space |
| CYP19A1 | cytochrome P450 family 19 subfamily A member 1 | 2,07E-10 | -3,616 | Cytoplasm |
| CD80 | CD80 molecule | 4,21E-16 | -3,617 | Plasma Membrane |
| SNAI1 | snail family transcriptional repressor 1 | 1,08E-16 | -3,728 | Nucleus |
| IFNG | interferon gamma | 1,48E-07 | -3,734 | Extracellular Space |
| CXCR5 | C-X-C motif chemokine receptor 5 | 6,52E-06 | -3,942 | Plasma Membrane |
| CCL2 | C-C motif chemokine ligand 2 | 3,88E-09 | -4,269 | Extracellular Space |
| BIRC3 | baculoviral IAP repeat containing 3 | 7,07E-12 | -4,475 | Cytoplasm |
| CD3G | CD3g molecule | 5,70E-12 | -4,609 | Plasma Membrane |
| KLK3 | kallikrein related peptidase 3 | 9,31E-09 | -4,697 | Extracellular Space |
| CD69 | CD69 molecule | 1,09E-10 | -4,744 | Plasma Membrane |
| NLRP2 | NLR family pyrin domain containing 2 | 3,55E-06 | -5,087 | Nucleus |
| IL6 | interleukin 6 | 6,94E-07 | -5,255 | Extracellular Space |
| ST8SIA1 | ST8 alpha-N-acetyl-neuraminide alpha-2,8-sialyltransferase 1 | 2,79E-14 | -5,281 | Cytoplasm |
| NOS1 | nitric oxide synthase 1 | 1,78E-14 | -5,291 | Cytoplasm |
| LBP | lipopolysaccharide binding protein | 6,54E-14 | -5,431 | Plasma Membrane |
| CXCL9 | C-X-C motif chemokine ligand 9 | 8,32E-07 | -5,503 | Extracellular Space |
| AICDA | activation induced cytidine deaminase | 5,94E-07 | -5,728 | Cytoplasm |
| CD40LG | CD40 ligand | 4,36E-15 | -6,115 | Extracellular Space |
| PTGDS | prostaglandin D2 synthase | 7,45E-14 | -6,952 | Cytoplasm |
| SCNN1A | sodium channel epithelial 1 subunit alpha | 2,42E-20 | -7,8 | Plasma Membrane |
| RAG2 | recombination activating 2 | 3,19E-09 | -8,342 | Nucleus |
| TFF3 | trefoil factor 3 | 1,70E-34 | -17,687 | Extracellular Space |
| APOD | apolipoprotein D | 5,63E-34 | -34,18 | Extracellular Space |
| CCL19 | C-C motif chemokine ligand 19 | 4,76E-18 | -50,332 | Extracellular Space |
